# Determinants of anti-PD-1 response and resistance in clear cell renal cell carcinoma

**DOI:** 10.1016/j.ccell.2021.10.001

**Published:** 2021-11-08

**Authors:** Lewis Au, Emine Hatipoglu, Marc Robert de Massy, Kevin Litchfield, Gordon Beattie, Andrew Rowan, Desiree Schnidrig, Rachael Thompson, Fiona Byrne, Stuart Horswell, Nicos Fotiadis, Steve Hazell, David Nicol, Scott T.C. Shepherd, Annika Fendler, Robert Mason, Lyra Del Rosario, Kim Edmonds, Karla Lingard, Sarah Sarker, Mary Mangwende, Eleanor Carlyle, Jan Attig, Kroopa Joshi, Imran Uddin, Pablo D. Becker, Mariana Werner Sunderland, Ayse Akarca, Ignazio Puccio, William W. Yang, Tom Lund, Kim Dhillon, Marcos Duran Vasquez, Ehsan Ghorani, Hang Xu, Charlotte Spencer, José I. López, Anna Green, Ula Mahadeva, Elaine Borg, Miriam Mitchison, David A. Moore, Ian Proctor, Mary Falzon, Lisa Pickering, Andrew J.S. Furness, James L. Reading, Roberto Salgado, Teresa Marafioti, Mariam Jamal-Hanjani, PEACE Consortium, Chris Abbosh, Kai-Keen Shiu, John Bridgewater, Daniel Hochhauser, Martin Forster, Siow-Ming Lee, Tanya Ahmad, Dionysis Papadatos-Pastos, Sam Janes, Peter Van Loo, Katey Enfield, Nicholas McGranahan, Ariana Huebner, Stephan Beck, Peter Parker, Henning Walczak, Tariq Enver, Rob Hynds, Ron Sinclair, Chi-wah Lok, Zoe Rhodes, David Moore, Reena Khiroya, Giorgia Trevisan, Peter Ellery, Mark Linch, Sebastian Brandner, Crispin Hiley, Selvaraju Veeriah, Maryam Razaq, Heather Shaw, Gert Attard, Mita Afroza Akther, Cristina Naceur-Lombardelli, Lizi Manzano, Maise Al-Bakir, Simranpreet Summan, Nnenna Kanu, Sophie Ward, Uzma Asghar, Emilia Lim, Faye Gishen, Adrian Tookman, Paddy Stone, Caroline Stirling, Nikki Hunter, Sarah Vaughan, Mary Mangwende, Lavinia Spain, Haixi Yan, Ben Shum, Eleanor Carlyle, Nadia Yousaf, Sanjay Popat, Olivia Curtis, Gordon Stamp, Antonia Toncheva, Emma Nye, Aida Murra, Justine Korteweg, Debra Josephs, Ashish Chandra, James Spicer, Ruby Stewart, Lara-Rose Iredale, Tina Mackay, Ben Deakin, Debra Enting, Sarah Rudman, Sharmistha Ghosh, Lena Karapagniotou, Elias Pintus, Andrew Tutt, Sarah Howlett, Vasiliki Michalarea, James Brenton, Carlos Caldas, Rebecca Fitzgerald, Merche Jimenez-Linan, Elena Provenzano, Alison Cluroe, Grant Stewart, Colin Watts, Richard Gilbertson, Ultan McDermott, Simon Tavare, Emma Beddowes, Patricia Roxburgh, Andrew Biankin, Anthony Chalmers, Sioban Fraser, Karin Oien, Andrew Kidd, Kevin Blyth, Matt Krebs, Fiona Blackhall, Yvonne Summers, Caroline Dive, Richard Marais, Fabio Gomes, Mat Carter, Jo Dransfield, John Le Quesne, Dean Fennell, Jacqui Shaw, Babu Naidu, Shobhit Baijal, Bruce Tanchel, Gerald Langman, Andrew Robinson, Martin Collard, Peter Cockcroft, Charlotte Ferris, Hollie Bancroft, Amy Kerr, Gary Middleton, Joanne Webb, Salma Kadiri, Peter Colloby, Bernard Olisemeke, Rodelaine Wilson, Ian Tomlinson, Sanjay Jogai, Christian Ottensmeier, David Harrison, Massimo Loda, Adrienne Flanagan, Mairead McKenzie, Allan Hackshaw, Jonathan Ledermann, Kitty Chan, Abby Sharp, Laura Farrelly, Hayley Bridger, George Kassiotis, Benny Chain, James Larkin, Charles Swanton, Sergio A. Quezada, Samra Turajlic, TRACERx Renal Consortium, Ben Challacombe, Ashish Chandra, Simon Chowdhury, William Drake, Archana Fernando, Karen Harrison-Phipps, Steve Hazell, Peter Hill, Catherine Horsfield, Tim O'Brien, Jonathon Olsburgh, Alexander Polson, Sarah Rudman, Mary Varia, Hema Verma

**Affiliations:** 1 Cancer Dynamics Laboratory, The Francis Crick Institute, London NW1 1AT, UK; 2 Renal and Skin Unit, The Royal Marsden NHS Foundation Trust, London SW3 6JJ, UK; 3 Cancer Immunology Unit, Research Department of Hematology, University College London Cancer Institute, London WC1E 6DD, UK; 4 Cancer Research UK Lung Cancer Centre of Excellence, University College London Cancer Institute, London WC1E 6DD, UK; 5 Cancer Evolution and Genome Instability Laboratory, The Francis Crick Institute, London NW1 1AT, UK; 6 Retroviral Immunology, The Francis Crick Institute, London NW1 1AT, UK; 7 Department of Bioinformatics and Biostatistics, The Francis Crick Institute, London NW1 1AT, UK; 8 Cancer Research UK Cancer Imaging Centre, Division of Radiotherapy and Imaging, The Institute of Cancer Research and Royal Marsden Hospital, London SW3 6JJ, UK; 9 Department of Pathology, the Royal Marsden NHS Foundation Trust, London SW3 6JJ, UK; 10 Department of Urology, the Royal Marsden NHS Foundation Trust, London SW3 6JJ, UK; 11 Department of Cellular Pathology, University College London Hospital, London NW1 2BU, UK; 12 Translational Immune Oncology Lab, Centre for Molecular Pathology, The Royal Marsden Hospital, Sutton SM2 5PT, UK; 13 Department of Pathology, Cruces University Hospital, Biocruces-Bizkaia Institute, 48903 Barakaldo, Bizkaia, Spain; 14 Department of Cellular Pathology, Guy’s & St Thomas’ NHS Foundation Trust, St Thomas’ Hospital, London SE1 7EH, UK; 15 Division of Research, Peter MacCallum Cancer Centre, Melbourne VIC 300, Australia; 16 Department of Pathology, GZA-ZNA Hospitals, Wilrijk, Antwerp, Belgium; 17 Cancer Metastasis Laboratory, University College London Cancer Institute, London WC1E 6DD, UK; 18 Department of Medical Oncology, University College London Hospitals, London NW1 2BU, UK; 19 Division of Infection and Immunity, University College London, London WC1E 6BT, UK; 20 University College London Cancer Institute, London WC1E 6DD, UK

**Keywords:** clear cell renal cell carcinoma, nivolumab, anti-PD-1, T cell receptor, TCR clonal maintenance, TCR clonal replacement, human endogenous retrovirus, multiregion, autopsy

## Abstract

ADAPTeR is a prospective, phase II study of nivolumab (anti-PD-1) in 15 treatment-naive patients (115 multiregion tumor samples) with metastatic clear cell renal cell carcinoma (ccRCC) aiming to understand the mechanism underpinning therapeutic response. Genomic analyses show no correlation between tumor molecular features and response, whereas ccRCC-specific human endogenous retrovirus expression indirectly correlates with clinical response. T cell receptor (TCR) analysis reveals a significantly higher number of expanded TCR clones pre-treatment in responders suggesting pre-existing immunity. Maintenance of highly similar clusters of TCRs post-treatment predict response, suggesting ongoing antigen engagement and survival of families of T cells likely recognizing the same antigens. In responders, nivolumab-bound CD8^+^ T cells are expanded and express GZMK/B. Our data suggest nivolumab drives both maintenance and replacement of previously expanded T cell clones, but only maintenance correlates with response. We hypothesize that maintenance and boosting of a pre-existing response is a key element of anti-PD-1 mode of action.

## Highlights

• 115 pre- and post-nivolumab multiregion tumor samples in a prospective phase II study
• Maintenance of pre-treatment expanded TCR clones associates with response
• Expanded CD8^+^ T cells upregulate GZMB/K in responders
• HERV expression reflects tumor purity and indirectly correlates with response

## Graphical abstract

**Figure:**
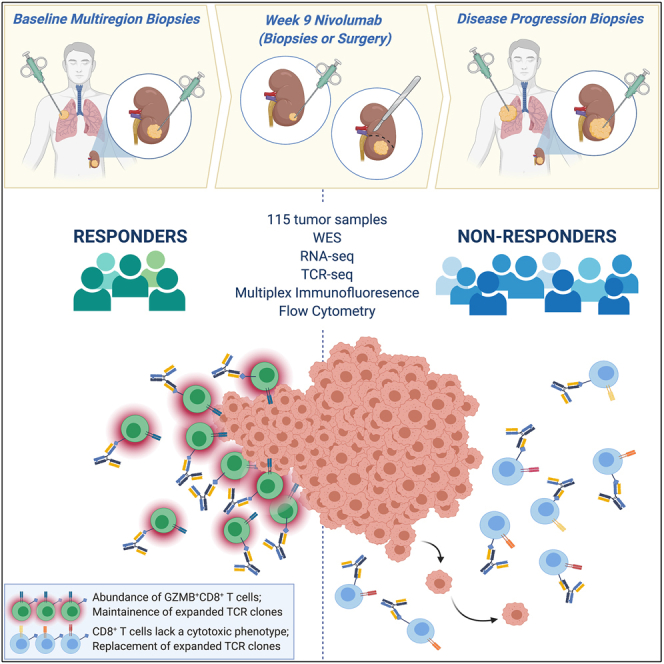

## Teaser

ADAPTeR is a phase II study of nivolumab (anti-PD-1) in treatment-naive patients with metastatic clear cell renal cell carcinoma. Through multi-omic analysis of multiregion tumor biopsies taken pre- and post-treatment, Au et al. evaluate genomic and tumor immune microenvironment features underpinning anti-PD-1 response and resistance using bulk and single-cell approaches.

## Introduction

Clear cell renal cell carcinoma (ccRCC) is the most common histological subtype of kidney cancer (Ricketts et al., 2018) with a rising global incidence (Smittenaar et al., 2016). Instances of spontaneous regression (Cole and Everson, 1956; Janiszewska et al., 2013; Snow and Schellhammer, 1982), and efficacy of interleukin-2 (Klapper et al., 2008; Rosenberg et al., 1989) and immune checkpoint inhibitors (CPI) (Motzer et al., 2015, 2018; Xu et al., 2020; Albiges et al., 2019) confirm ccRCC as an immunogenic tumor type, though the nature of the antigenic stimulus remains unknown. ccRCC carries a modest tumor mutational burden (TMB) (median of 1.42 mutations per megabase [mut/mb]) (de Velasco et al., 2016), 10-fold lower than melanoma and comparable to immune “cold” tumors (Alexandrov et al., 2013). In contrast to melanoma (Snyder et al., 2014), non-small cell lung cancer (Rizvi et al., 2015; Hellmann et al., 2018), bladder (Aggen and Drake, 2017), and colorectal cancers (Le et al., 2015), TMB does not associate with CPI response in ccRCC (Braun et al., 2020; McDermott et al., 2018; Motzer et al., 2019). ccRCC is enriched for frameshift insertion and deletions (fsINDELs) (Turajlic et al., 2017), which can generate novel open-reading frames triggering a large number of highly distinct neoantigens. However, so far, fsINDEL burden has not been shown to predict benefit from CPI in ccRCC (Braun et al., 2020; McDermott et al., 2018; Motzer et al., 2019), again in contrast to other tumor types (Turajlic et al., 2017; Litchfield et al., 2020). Finally, an association between mutations in PBRM1, present in ∼60% of ccRCC, and response to CPI has been reported (Braun et al., 2019, 2020; Miao et al., 2018), though the association has not been observed consistently (McDermott et al., 2018; Motzer et al., 2019, 2020a; Motzer et al., 2019, Abou Alaiwi et al., 2020; Motzer et al., 2020a).

Large-scale tumor transcriptome analyses show ccRCCs to be among the most highly immune-infiltrated solid tumor types (Ricketts et al., 2018; Rooney et al., 2015), but in contrast to other cancers, high immune infiltration correlates with poor outcomes following nephrectomy (Fridman et al., 2017). In the context of treatment with CPI, high T cell/low myeloid infiltration and high B cell abundance are reported to be enriched in responders to atezolizumab (anti-PD-L1) (McDermott et al., 2018) and nivolumab (anti-PD-1) (Helmink et al., 2020), respectively. However, cross-validation of these features as predictive biomarkers has yielded inconsistent findings (Bi et al., 2021; Motzer et al., 2020a, 2020b; Braun et al., 2020), potentially owing to immune intratumor heterogeneity (ITH) (Gulati et al., 2014; Braun et al., 2021), especially as prior studies have relied on single tumor region evaluation. Our group has previously shown that ITH is a frequent feature of ccRCC that associates with patterns of metastatic spread and outcomes following surgery (Gerlinger et al., 2014; Turajlic et al., 2018a, 2018b). As such, ITH complicates evaluation of prognostic and predictive biomarkers in all settings and requires due consideration.

**Figure 1:**
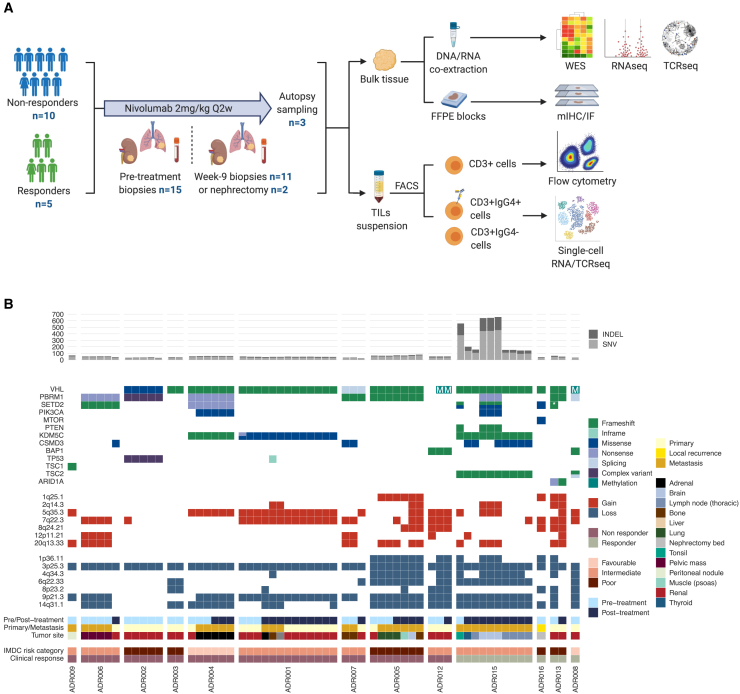
Experimental workflow, patients and samples overview, and genomic characteristics of the ADAPTeR cohort (A) Overview of experimental workflow. The numbers (n) of patients contributing to sample collection at different timepoints are shown. (B) Heatmap of WES analysis demonstrating nsSNV and INDEL burden, somatic driver alterations annotated with pre/post-treatment, tumor site, IMDC risk category, and nivolumab response. Composite mutations are annotated with dual colors. Composite mutations (two or more non-synonymous somatic mutations in the same gene and tumor sample [Gorelick et al., 2020]) involving *SETD2*, *KDM5C*, and *TSC2* are shown. Complex mutations in ADR002: *PBRM1* frameshift insertion chr3:52584573:->T and non-frameshift deletion chr3:52584576:TAT>-; *TP53* missense mutation chr17:7572969:A>T and frameshift insertion chr3:7572962:->CT. ^∗^Denotes two distinct fsINDEL mutations in one tumor sample in ADR013. See also Figures S1, S2, Tables S1, and S2.

Our report concerns ADAPTeR (NCT02446860), a phase II, single-arm, open-label study of nivolumab in treatment-naive patients with metastatic ccRCC. Patients underwent multiregional tumor sampling of primary and/or metastatic sites at baseline, week 9, at surgery (if performed), and disease progression. A key aim of ADAPTeR was to evaluate molecular and tumor immune microenvironment (TME) features throughout therapy. In addition, patients were co-recruited to TRACERx Renal (TRAcking Cancer Evolution through therapy[Rx]; NCT03226886), and PEACE (Posthumous Evaluation of Advanced Cancer Environment; NCT03004755) studies to expand the spatial and temporal breadth of profiling. We present an integrated analysis of response to nivolumab and whole-exome and RNA sequencing (RNA-seq), TCR profiling, and immunohistochemistry/multiple immunofluorescence (IHC/mIF); as well as high-dimensional flow cytometry across longitudinal, multiregion fresh tumor samples in this cohort (Figure 1A).

## Results

### Patient characteristics and clinical benefit to nivolumab

Fifteen patients were enrolled from October 2015 to June 2018. Demographic and clinical characteristics are shown in Table S1. Thirteen (87%) patients had intermediate- or poor-prognostic risk disease as defined by International Metastatic RCC Database Consortium risk categorization (IMDC) (STAR Methods) (Heng et al., 2009). At clinical data lock (December 2018), median follow-up was 12.5 (range, 3.9 to 27.3) months. Six deaths occurred, all due to progressive disease. The median progression-free (PFS) and overall survival (OS) were 4.1 and 22.2 months, respectively. For translational analyses, we defined patients who derived clinical benefit (hereon termed “responders”) as those who had a partial response (PR) or stable disease (SD), as measured by Response Evaluation Criteria In Solid Tumors (STAR Methods) for ≥6 months (five patients). Patients who derived minimal clinical benefit (hereon termed “non-responders”) were classified by progressive disease within 6 months of enrollment regardless of best response (10 patients). Five patients (33%) had a PR, of whom one patient (ADR005) had short-lived PR (<6 months, classified as non-responder). Six patients (40%) had SD, of which one patient (ADR011) had durable (>6 months) SD (classified as responder) (Figure S1A; Table S1). Two patients underwent a cytoreductive nephrectomy during the study. We observed no association between age, sex, IMDC risk category, and/or presence of sarcomatoid/rhabdoid features (n = 2) and response to nivolumab (Table S1). Overall, these clinical data are consistent with a larger phase II (n = 110) cohort study of first-line pembrolizumab in patients with ccRCC (McDermott et al., 2021).

### Tumor molecular features do not correlate with nivolumab response

All patients underwent image-guided percutaneous tumor biopsies with additional archived and fresh samples collected via TRACERx Renal and PEACE studies. Fifteen patients had pre-treatment biopsies, and 13 patients had post-treatment biopsies. In total, 115 tumor samples (fresh and archived) were available for translational analyses (see Figure S1A for consort diagram; Table S2 for sample characteristics). Eighty-one fresh tumor samples and matched germline DNA underwent whole-exome sequencing (WES). Subsequently, 22 samples were excluded: 21 due to low tumor purity, which is expected with image-guided biopsies, and one excluded due to sample contamination. Fifty-nine tumor samples from 13 patients were of sufficient quality for downstream mutation analyses (STAR Methods).

Median sequencing depth was 199x (range 130–359x) (Table S2). Neither pre-treatment TMB (median 0.9 mut/mb; range 0.4–11.1), fsINDEL load (median 9; range 0–169), nor expressed non-synonymous single nucleotide variants (nsSNVs) or fsINDELs associated with response to nivolumab (Figure S1B). Post-treatment, we found no evidence of stronger depletion of mutations (nsSNVs or fsINDELS) that encode for neoantigens compared with the remaining non-synonymous mutations (Figure S1C). Molecular features of this cohort were typical of ccRCC (Ricketts et al., 2018; Turajlic et al., 2018b), including mutations in *VHL* (77%), *VHL* methylation in an additional 15%, *PBRM1* (62%), *SETD2* (38%), *BAP1* (15%), and *KDM5C* (38%), with both clonal and subclonal alterations detected (Figure 1B). There was no association between mutations in any gene and response to nivolumab. Copy number landscape was also typical of ccRCC with clonal loss of 3p25.3 detected in all tumors and 9p21.3 and/or 14q31.1 loss observed in 12 of 13 patients, consistent with our previous findings in metastatic ccRCC (Turajlic et al., 2018a) (Figure 1B). Weighted genome instability index (wGII) as a global measure of chromosomal complexity was not predictive of nivolumab response (p = 0.076) (Figure S1B; STAR Methods). We previously showed that ITH index, a metric developed in the context of *ex vivo* multiregion sampling, was prognostic in ccRCC (Turajlic et al., 2018b). In ADAPTeR, we found no association of ITH index and response to nivolumab (p = 0.88); however, ITH is likely to be underestimated in this study (STAR Methods). No driver somatic copy number alterations (SCNAs) associated with response.

Intermetastatic heterogeneity, which can underpin differential therapy response (Birkeland et al., 2018; Sakamoto et al., 2020; Sveen et al., 2016; Sebagh et al., 2016), was evaluated through postmortem sampling in three cases. Of particular interest were the findings in ADR015. This was a patient with stage IV disease upon enrollment into ADAPTeR, involving surgical bed recurrence, bone metastases, and nodal disease, with a tonsillar metastasis resected pre-treatment. PFS on nivolumab was 8.4 months (overall “responder”; best response was SD evident at all sites), with disease progression in the brain resulting in death 27.3 months after trial enrollment. All metastatic deposits, including an incidental thyroid metastasis, were sampled at postmortem and whole-exome sequenced. We found evidence of genetic divergence between disease sites that progressed (brain) and responded (nodal metastases) under nivolumab. Uncharacteristically high TMB (median 10.8 mut/Mb) and fsINDEL load (median 166), and therefore a high predicted neoantigen load, was evident in the progressive brain and resected treatment-naive tonsillar metastases, but not in treatment-responsive disease sites (median TMB 1.3mut/Mb; fsINDEL load 8) (Figure S2A; Table S2). Most of the excess mutations were contributed by C > T at GpCpN trinucleotides (Signature 15), which result from defective DNA mismatch repair (MMRD) (Alexandrov et al., 2013). Accordingly, we detected biallelic inactivation of *MLH1* (pathogenic mutation(ClinVar) with concurrent loss of heterozygosity [LOH] through canonical 3p loss, as *MLH1* is encoded at 3p22.2) in resistant, but not the nivolumab-sensitive sites (STAR Methods). *MLH1* loss leads to accumulation of a high number of mutations (Kloor and von Knebel Doeberitz, 2016), and is associated with better response to CPI (Le et al., 2017). However, the sites with MMRD characterized by nivolumab resistance, but not the nivolumab-sensitive sites, also harbored a beta-2-microglobulin (*B2M*) mutation with LOH (Figure S2A; STAR Methods), which can lead to loss of antigen presentation (Doherty, 1995). We confirmed loss of *MLH1* and *B2M* protein expression by immunohistochemistry (IHC) in resistant metastatic sites and in a single area of the primary tumor resected 5 years before study entry (Figure S2B). Taken together, it appears that subclonal loss of *MLH1* led to accumulation of excess neoantigens, and subsequent loss of antigen presentation presumably due to immune selective pressure. This tumor subclone was represented in nivolumab-resistant metastases, reconciling the mixed treatment response observed in this case. In ccRCC, MMRD has been reported, albeit infrequently (Altavilla et al., 2010). However, while loss of *B2M* as a mechanism of CPI resistance has been described in other tumor types (Zaretsky et al., 2016; Gettinger et al., 2017), this has not been described to date in ccRCC.

### ccRCC-specific human endogenous retrovirus expression reflects tumor purity and associates with lack of response to anti-PD-1

Prior reports have indicated that the presence of intratumoral cytotoxic T cells (Rooney et al., 2015) and response to nivolumab (Panda et al., 2018; Smith et al., 2018; Ficial et al., 2020) in ccRCC are associated with tumoral expression of human endogenous retroviruses (HERVs), suggesting they may provide a source of cancer-specific antigens. Indeed, T cell targeting of a member of the HERVE family has been demonstrated to mediate regression of kidney cancer in a stem cell transplant recipient (Takahashi et al., 2008). We therefore examined if the outcome of nivolumab in the ADAPTeR cohort was associated with HERV expression patterns, as determined by RNA-seq analysis. To this end, we performed RNA-seq on 60 tumor samples, 33 pre-treatment and 27 post-treatment (week 9), representing 14 patients (see Figure S1A for consort diagram; Table S2 for sample characteristics; STAR Methods).

Prior studies (Rooney et al., 2015; Panda et al., 2018) used a limited set of 66 HERV loci annotated by Mayer et al. (2011) or 3,173 HERV loci (Smith et al., 2018) annotated by Vargiu et al. (2016). To allow direct comparison between these two previous annotations, as well as with a more complete HERV annotation, we first updated the Vargiu et al. annotation, which was based on an earlier release of the human genome (GRCh37) to the current release (GRCh38), and compared the coordinates of unique elements in both annotations to a complete custom repeat region annotation we previously built (Attig et al., 2017) (STAR Methods). This comparison revealed major discrepancies that may have affected prior analyses. For example, HERV loci considered as a single integration in our custom annotation were fragmented in the Mayer et al. and/or Vargiu et al. annotations, and vice versa (Table S3). Further, we found prior HERV annotations that were either incomplete or extended beyond integration boundaries to include exons of adjacent genes belonging to separate transcription units (Figure S3A). Such discrepancies affected HERV integrations previously associated with immune response in ccRCC (e.g., ERV3-2 and ERVK-10) (Rooney et al., 2015; Smith et al., 2018) (Figure S3A). Accounting for the above discrepancies, the previously annotated 66 and 3,173 HERVs corresponded to 7,989 repeat loci in our custom annotation (Table S3).

**Figure 2:**
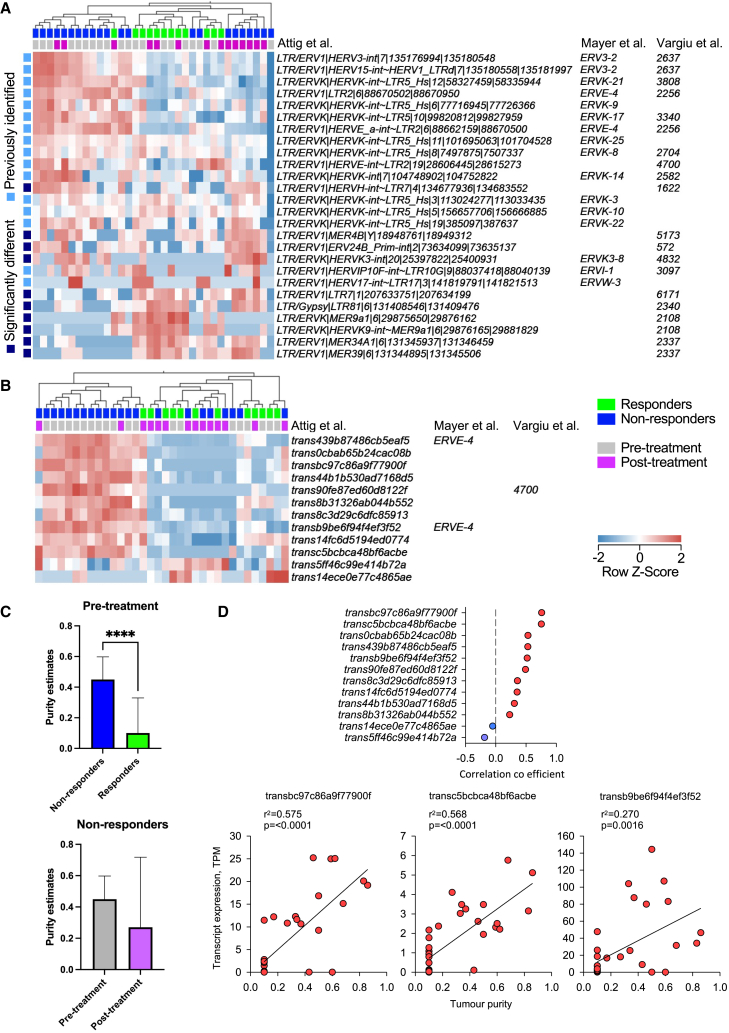
Expression of HERVs and LTR-overlapping transcripts in ccRCC according to tumor purity (A) Hierarchical clustering of patient samples according to the relative expression of HERVs previously associated with cytotoxic T cell presence, response to immunotherapy, or the provision of antigenic epitopes. (B) Hierarchical clustering patient samples according to the 12 LTR-overlapping transcripts that were differentially expressed (≥2-fold change, q ≤ 0.05) between responders and non-responders or affected by nivolumab. (C) Comparisons of tumor purity. Median values are shown; top whiskers indicate range from third quartile to maximum. ^∗∗∗∗^p < 0.0001; Mann-Whitney *U* test. (D) Distribution plot of significant Spearman’s rank-order correlation between tumor purity and TPM expression of the 12 HERVs differentially expressed between responders and non-responders. See also Figure S3 and Table S3.

None of the HERV loci previously associated with cytotoxic T cell presence, ccRCC response to CPI, or the provision of antigenic epitopes (Rooney et al., 2015; Panda et al., 2018; Smith et al., 2018; Takahashi et al., 2008) were differentially expressed between responders and non-responders or were affected by immunotherapy in this cohort (Figure 2A). Moreover, none of the previously tested 7,989 HERV annotations were affected by immunotherapy. However, 10 HERV annotations, from eight distinct loci within this limited list, distinguished responders from non-responders, either pre- or post-treatment (≥2-fold change, q ≤ 0.05), and half of them appeared restricted to responders pre/post-treatment and non-responders post-treatment (Figure 2A). Thus, our analysis revealed a different pattern of HERV association with the outcome of ccRCC immunotherapy than previously reported by others (Panda et al., 2018; Smith et al., 2018).

To investigate possible reasons of the observed association, we re-examined tumor-cell intrinsic expression of the selected HERVs. Many of the significantly differentially expressed HERV loci, including those previously associated with anti-tumor T cell responses (Rooney et al., 2015; Panda et al., 2018) such as ERV3-2, were not specific to ccRCC and were highly expressed in purified immune cells (Figure S3B) (STAR Methods). For example, the LTR/ERVK|HERVK9-int∼MER9a1|6|29876165|29881829 integration within the HLA locus is expressed in most immune cell subsets and the LTR/ERV1|LTR7|1|207633751|207634199 integration is expressed in neutrophils (Figure S3B). Of note, HERVs found to be expressed in immune cells were enriched for members of the HERVK group (Figure S3B). It was, therefore, likely that association between HERVK with responders in this study and cytotoxic T cell presence previously (Rooney et al., 2015; Panda et al., 2018), resulted from high expression in immune cells. In contrast, HERVs that were not expressed in immune cells, such as the previously identified ERVE-4 (Rooney et al., 2015) and HERV 4700 (Smith et al., 2018), were expressed at higher levels in pre-immunotherapy non-responders (Figure 2A). One exception was ERV3-2, which was also expressed at higher levels pre-treatment in non-responders, despite also showing the highest expression in immune cells, particularly neutrophils (Figures 2A and S3B). Therefore, the association between HERV expression in bulk tumor RNA-seq data and CPI responses may, in fact, reflect the level and type of immune infiltration (which, in itself, is linked with the response [McDermott et al., 2018; Motzer et al., 2020b]).

To overcome the limitations of genomic HERV annotations, we next quantified HERV expression in the ADAPTeR cohort using a *de novo* assembled cancer transcriptome (Attig et al., 2019), and focused on ccRCC-specific HERVs. This method takes into consideration the structure of transcripts that overlap with repeat elements, which allows for more accurate quantification using transcript per million (TPM) calculations (Attig et al., 2019). Using this method, we previously identified 570 *de novo* assembled transcripts overlapping with LTR elements that were highly specific for ccRCC (Attig et al., 2019). The majority of these transcripts were expressed (≥0.5 TPM) in the majority of the ADAPTeR samples, but only 12 of them, from nine distinct loci, were differentially expressed (≥2-fold change, q ≤ 0.05) between responders and non-responders or were affected by nivolumab (Figure 2B). Importantly, almost all of them were expressed predominantly in non-responders pre-treatment and included the members of the HERVE group (ERVE-4 and HERV 4700) that were previously associated with anti-tumor T cell responses in ccRCC (Rooney et al., 2015; Smith et al., 2018; Takahashi et al., 2008) (Figure 2B). Thus, the use of a complete transcript assembly and TPM calculations, as opposed to normalized reads used previously, further supported the association of ccRCC-specific LTR elements with lack of response to anti-PD-1.

Collectively, these data suggest that transcription of HERVs and other LTR elements that are highly specific to ccRCC were overexpressed in non-responders pre-treatment and were associated with an absence of ongoing anti-tumor immune responses and lack of response to anti-PD-1. As these LTR elements were selected for their specificity in ccRCC and lack of expression in other cell types, their elevated transcription in non-responders pre-treatment likely reflects higher tumor purity (i.e., lower immune infiltration) compared with responders (Figure 2C). Post-treatment, ccRCC-specific HERV expression in non-responders normalized relative to responders, consistent with a reduction in tumor purity likely due to immune infiltration in non-responders (Figures 2B and 2C). Accordingly, expression of ccRCC-specific LTR element-overlapping transcripts correlated with tumor purity (Figure 2D). In summary, while these data do not exclude the provision of antigens or direct modulation of the immune response, they suggest that the association of HERV expression with CPI response reflects the cellular composition in bulk samples in ccRCC.

### Nivolumab induces T cell activation and upregulation of TCR signaling in responders

**Figure 3:**
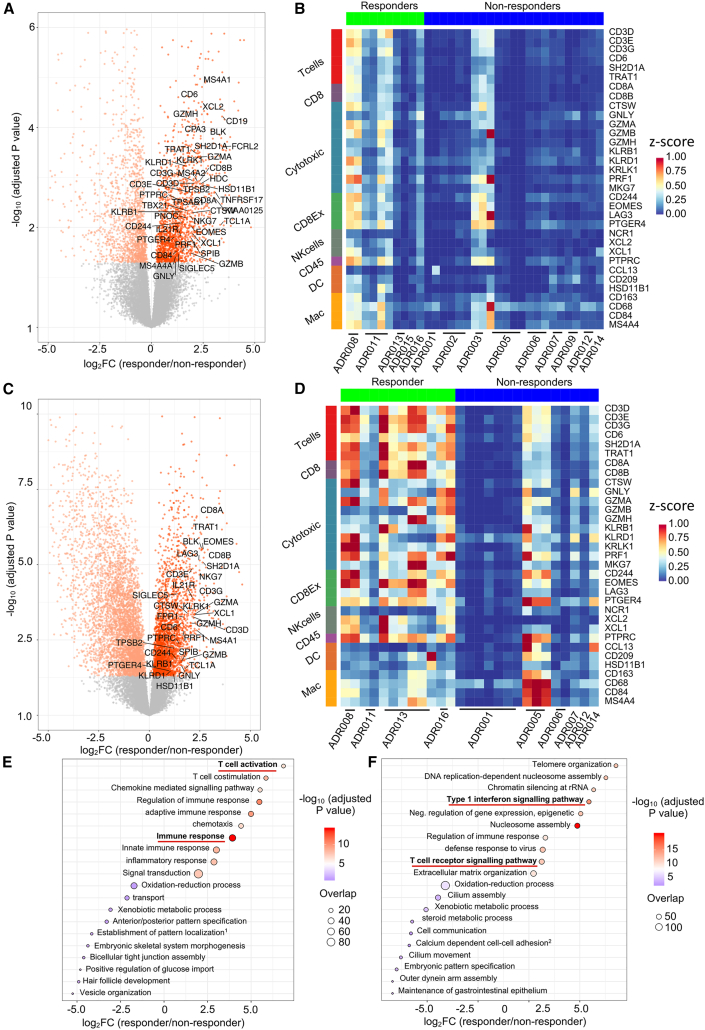
GSEA and immune deconvolution by RNA-seq shows higher levels of immune infiltration and activation in responders compared with non-responders under nivolumab (A) Transcripts differentially regulated pre-treatment between responders and non-responders (n = 33 samples, 14 patients, negative binomial Wald test, Benjamini-Hochberg corrected p values). A total of 3,382 transcripts were differentially regulated (false discovery rate [FDR] <0.05); the ones that overlap with the Danaher immune score gene list are labeled. No differentially regulated genes were downregulated between response groups, hence the left side of the plot appears unannotated. (B) Heatmap showing the relative expression (*Z* scores) of genes from eight Danaher immune modules in pre-treatment samples. (C) Transcripts differentially regulated post-treatment between responders and non-responders (n = 27 samples, 10 patients, negative binomial Wald test, Benjamini-Hochberg corrected p values). A total of 7,975 transcripts were differentially regulated (FDR <0.05); the ones that overlap with the Danaher immune score gene list are labeled. No differentially regulated genes were downregulated between response groups, hence the left side of the plot appears unannotated. (D) Heatmap showing the relative expression (*Z* scores) of genes from eight Danaher immune modules in post-treatment samples. (E) GOBP pathway analysis of genes preferentially upregulated and downregulated pre-treatment in responders, Overlap (n), number of significant genes from a pathway (hypergeometric test). (F) Gene ontology biological process (GOBP) pathway analysis of genes preferentially upregulated and downregulated post-treatment in responders, Overlap (n), number of significant genes from a pathway (hypergeometric test). See also Figures S4 and S5.

Next, we performed differential gene expression, gene set enrichment (GSEA), and immune subset deconvolution pre- and post-nivolumab (STAR Methods). Tumors from responders harbored significantly higher levels of T cells (based on Danaher signature [Danaher et al., 2017]) both pre- and post-treatment compared with non-responders (p = 0.019 and p = 0.038, respectively), but T cell infiltration increased on-treatment irrespective of response (Figures 3A–3D and S4). We found higher expression of CD3E, CD8A, Granzyme B (GZMB), and TCF7, in responders compared with non-responders, particularly post-treatment (Figure S4). “Immune-activation” and “TCR signaling” pathways were enriched in responders but not non-responders (Figures 3E and 3F).

Immune heterogeneity has been reported in ccRCC (Gulati et al., 2014; Braun et al., 2021; Krishna et al., 2021) but not evaluated in the context of treatment. Of the 12 patients who contributed multiple samples at a single time point, three presented a mixture of immune “hot” and “cold” biopsies at the given time point (Figure S5). For example, in ADR005 (non-responder) pre-treatment, one biopsy from primary tumor was immune “hot” and four (two from primary tumor and two from a lung metastasis) were immune “cold.” Post-treatment, two biopsies (representative of previously “cold” lung metastasis) were immune “hot,” consistent with nivolumab-induced immune infiltration. In ADR013 (responder), longitudinal sampling of the primary tumor showed the two pre-treatment biopsies were immune “cold,” while post-treatment, five biopsies were “hot” and one was “cold” (Figures 3B, 3D, and S5). On review of hematoxylin and eosin (H&E) images, the one immune “cold” post-treatment biopsy was mostly necrotic, likely reflecting nivolumab response. These two cases demonstrate that immune heterogeneity is both inherent to ccRCC pre-treatment and altered by CPI and response post-treatment. ADR003 was the only case with consistently immune “hot” baseline biopsies by RNA-seq yet was a non-responder. Review of H&E revealed distinct immune “deserted” and heavily infiltrated areas within a single sample. In this case, it remains possible that clones evading immune recognition/infiltration, unaccountable by bulk-RNA-seq, may have driven the patient's outcome. Taken together, these examples highlight challenges in patient stratification by immune infiltration status in ccRCC, especially with single-sample approaches.

Finally, we evaluated the association between published gene expression signatures and nivolumab response (STAR Methods). IMmotion150 study T_eff_^high^ signature (McDermott et al., 2018), but not T_eff_^high^/Myeloid^low^ signature was enriched in responders compared with non-responders (p = 0.042 and p = 0.038 pre- and post-treatment, respectively) (Figure S4). The 26-gene Javelin101 signature (Motzer et al., 2020b) was also enriched in responders compared with non-responders (p = 0.028 and p = 0.038 pre- and post-treatment, respectively). Cross-validation of these gene expression signatures in other single-sample studies have yielded inconsistent findings (Motzer et al., 2020a, 2020b; Braun et al., 2020; Krishna et al., 2021). In contrast, the signatures performed consistently in our multiregion cohort, despite inherent differences across studies in treatment regimens and type of tissue that was profiled.

### CD8^+^ T cells upregulate GZMB following nivolumab in responders

Next, to evaluate dynamic TME changes under nivolumab with greater resolution, we applied antibody panels (immunohistochemistry [IHC] and multiplex immunofluorescence [mIF]; STAR Methods) focused on T cells, macrophages (McDermott et al., 2018; Bi et al., 2021), B cells, and plasma cells (Helmink et al., 2020; Petitprez et al., 2020) to 61 formalin-fixed paraffin-embedded tumor samples (41 pre-treatment; 20 post-treatment) from 14 patients (Figure S1A; STAR Methods).

**Figure 4:**
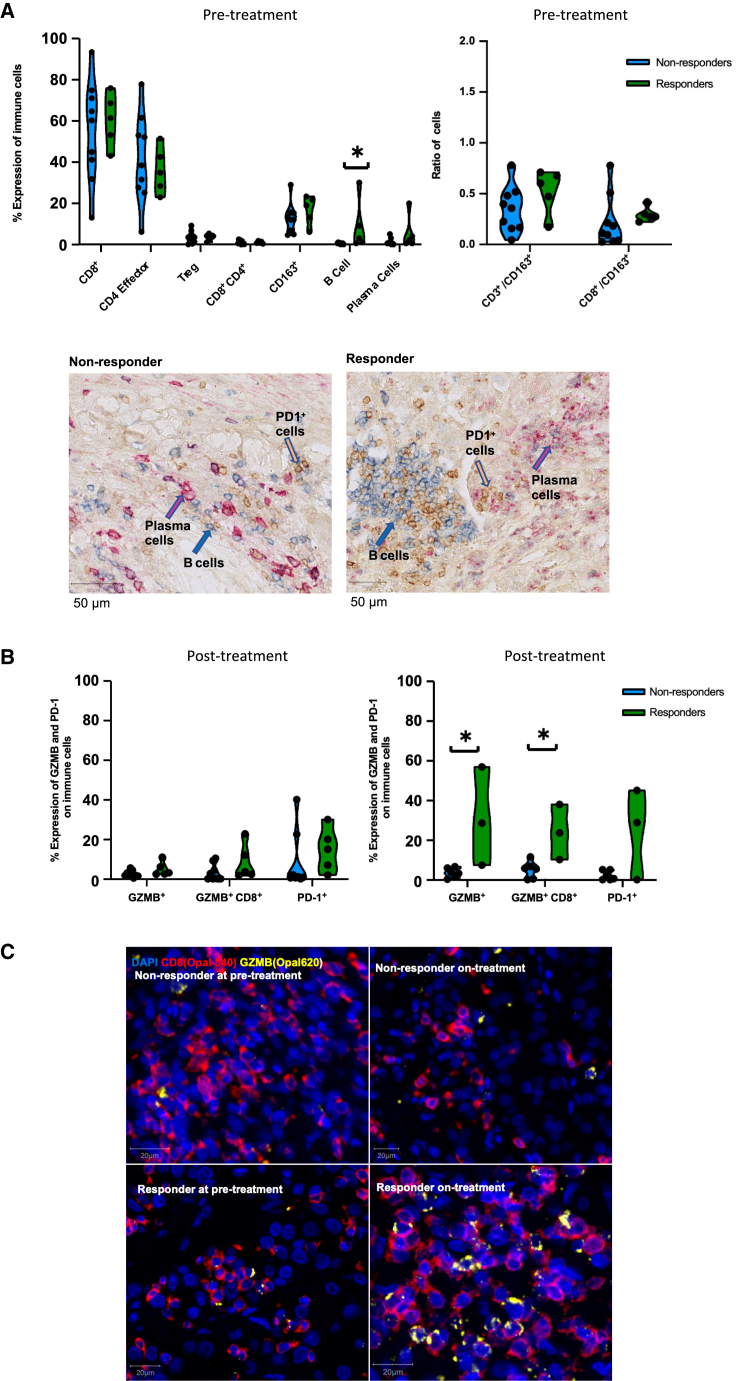
Quantification and immunophenotyping of pre- and post-treatment infiltrating immune cells by IHC and mIF (A) Comparison of T cell subset (out of total T cells), CD163^+^ myeloid cells, B cell and plasma cell infiltration in treatment-naive samples in responders (n = 5) and non-responders (n = 9) is shown on the left. On the right is the ratio between CD3^+^ (total T cells) and CD163^+^ myeloid cells and CD8^+^ and CD163^+^ cells at baseline. B cell and plasma cell scoring was done by using IHC. Other markers were scored by using IF. IHC images of representative responder and non-responder patients pre-treatment showing B cell (blue), PD-1^+^ cells (yellow), and plasma cells (magenta) infiltration. (B) Level of overall GZMB, GZMB^+^CD8^+^, and overall PD-1 expression in responders and non-responders in treatment-naive and on-treatment samples is shown. PD-1 staining was performed with IHC. All other markers were stained with IF. (C) mIF images showing GZMB^+^CD8^+^ cells in a representative responder and non-responder patient at baseline and post-nivolumab treatment. Median values were used for each patient and a two-sided Mann-Whitney *U* statistical test was used for the analysis. ^∗^p < 0.05. See also Figure S6.

We observed no difference in T cell number (CD8^+^, CD4^+^, CD8^+^CD4^+^, or T regulatory cells [Tregs]), CD8^+^/Treg and CD4^+^effector/Treg ratio, or total PD-1 expression between response groups, at any time point (Figures 4A, 4B, and S6A–S6C). Low levels of GZMB expression were observed prior to treatment in both responders and non-responders; however, post-treatment (week 9), both overall (p = 0.024) and CD8^+^ T cell-specific GZMB expression (p = 0.047) significantly increased in responders compared with non-responders (Figures 4B, 4C, and S6D). The level of CD163^+^ myeloid cells alone or as a ratio to T cells (CD3^+^/CD163^+^ and CD8^+^/CD163^+^) did not associate with response (Figures 4A and S6C). We observed significantly more B cells in responders (p = 0.02) (Figure 4A) at baseline, consistent with prior reports (Helmink et al., 2020), but not on-treatment. There were no differences in the number of plasma cells between response groups at any time point (Figures 4A and S6A).

We note observations made from bulk RNA-seq and IHC/mIF data showed trends that were in the same direction but did not always reach statistical significance in some instances. For example, increased B cells and higher GZMB expression in responders was evident by both IHC/mIF and RNA-seq (Figure S4), but only statistically significant by IHC/mIF. CD4^+^/8^+^ T cell numbers and PD-1 expression were not statistically different by IHC/mIF between response groups but were significantly enriched in responders by RNA-seq. These findings reflect the known imperfect correlation between protein and mRNA levels for many genes and limitations of immune classification by bulk RNA-seq (Newman et al., 2015; Braun et al., 2020), as compared with the single-cell resolution afforded by histology-based methods.

### Maintenance of previously expanded TCR clones and CDR3 clustering supports ongoing antigen-driven stimulation of pre-existing T cells in responders

The question of whether tumor-specific T cells activated by CPI pre-exist in the tumor or are replaced by new T cell clones recruited to the TME remains under debate (Riaz et al., 2017; Cha et al., 2014; Wu et al., 2020; Li et al., 2019b) and has not been investigated in the context of ccRCC. Crucially, this question can only be addressed with paired pre- and post-treatment samples, such as those in ADAPTeR. We sequenced the β-chain TCR repertoires from 14 patients pre- and post-treatment, including 64 tumor and 29 peripheral blood mononuclear cell (PBMC) samples (Figure S1A; STAR Methods). To quantify TCR heterogeneity within each patient, described in other cancer types (Joshi et al., 2019; Zhang et al., 2018a; Angelova et al., 2018), we performed pairwise comparison of TCR repertoires of multiple samples from each time point for each patient (STAR Methods). TCR repertoire similarity varied, from near-complete concordance between biopsies in some patients, to minimal overlap in others (Figures S7A and S7B). To mitigate against the effects of TCR heterogeneity in the cohort-level analysis, we pooled TCR sequences from multiple tumor regions taken at each time point for each patient.

**Figure 5:**
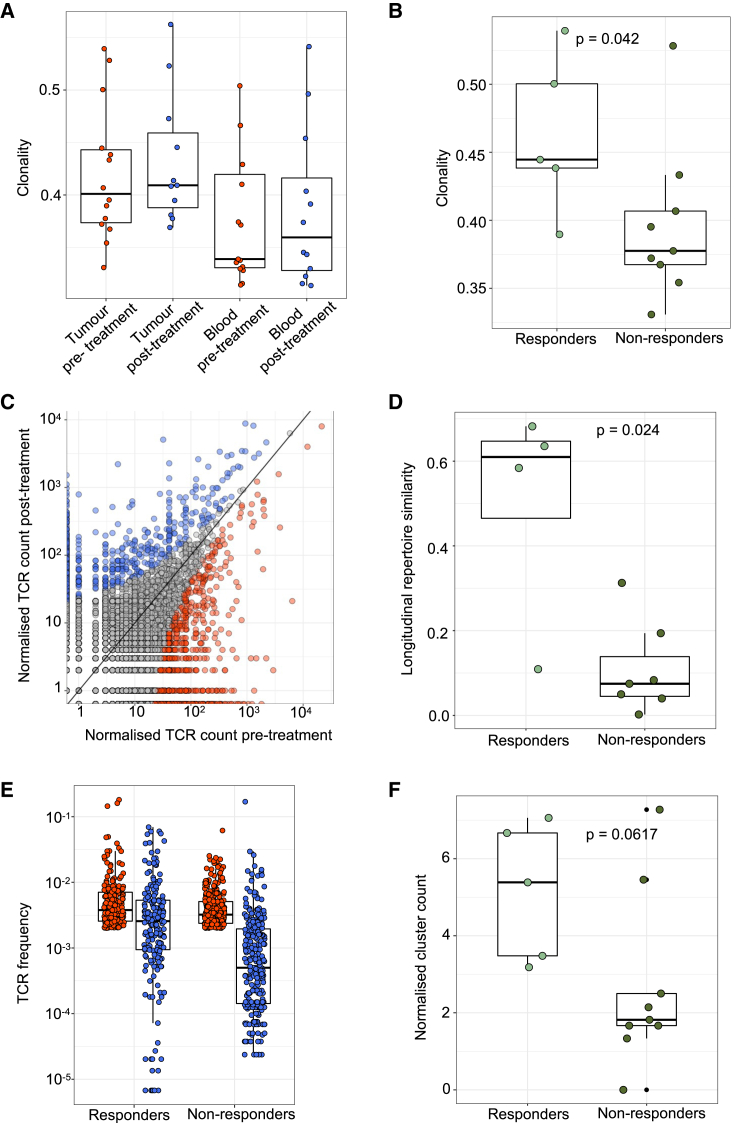
TCR-seq demonstrates maintained clonal expansion through persistent antigenic stimulation associate with nivolumab response (A) The intratumoral and peripheral TCR repertoire clonality scores are shown for each patient at each time point. (B) The intratumoral TCR repertoire clonality scores pre-treatment are shown for each patient, categorized by response to nivolumab. Mixed-effect model p value shown. (C) Correlated clone sizes in tumor samples. Scatterplots of tumor clone size pre- and post-treatment are shown for all patients. Clones are colored by expansion/contraction status (STAR Methods). (D) The intratumoral similarity (cosine) scores between pre-treatment (red) and on-treatment (blue) are shown for each patient (n = 12). Patients are split between responders and non-responders. Responding patients exhibit greater cosine score, with the two-sided Mann-Whitney test p value shown. (E) The frequency distribution of the intratumoral expanded TCRs pre-treatment (red circles; n = 469 individual TCRs combined from 12 patients) and post-treatment (blue circles). Only TCRs that were detected post-treatment were included. (F) The clustering algorithm was run on all patients, and the pre-treatment normalized number of clusters for the networks containing expanded sequences is shown. Two-sided Mann-Whitney test p value shown; n = 14 patients. The minimum and maximum are indicated by the extreme points of the box plot; the median is indicated by the thick horizontal line; and the first and third quartiles are indicated by box edges. See also Figures S7–S9 and Table S4.

Cohort-wide, the median number of unique β-chain transcripts in tumor and blood samples was 3,644 and 21,370, respectively. We quantified TCR clonality through a “repertoire clonality score,” where low scores correlate with more diverse repertoire and high scores with expansion of dominant TCR clones (STAR Methods). Overall, TCR clonality was higher in tumor samples compared with PBMCs (Figure 5A), likely reflecting intratumoral clonal expansion. We observed higher baseline intratumoral TCR clonality in responders compared with non-responders (p = 0.042) (Figure 5B), but post-treatment the difference was not significant (p = 0.25) (Figure S7C). Peripheral TCR clonality was not associated with response at any time point (Figure S7D). The number of clonotypes that increased in frequency (“expansion”) or decreased in frequency (“contraction”) post-treatment were not significantly different between response groups, intratumorally or peripherally (Figures 5C, S7E, and S7F).

Next, we computed a cosine score that reflected how similar TCR repertoires were pre- and post-treatment (STAR Methods), to evaluate the link between nivolumab response and maintenance of pre-existing or replacement with novel TCR clonotypes. Tracking the total TCR repertoires, we observed a greater degree of TCR clonal maintenance in responders (greater TCR repertoire similarity between timepoints) compared with non-responders intratumorally (p = 0.024) (Figure 5D), but not in PBMCs (Figure S7G). In particular, pre-existing *expanded* TCR clones were more likely to be maintained in responders compared with non-responders, where they were frequently replaced (p = 0.024, Figures 5E and S8A). The appearance of novel expanded T cell clones post anti-PD-1 did not correlate with response to nivolumab (Figure S7E).

Given the broader debate around TCR clonal dynamics and CPI response, we reanalyzed longitudinal TCR-sequencing (TCR-seq) data from a study by Yost et al. (2019) (see Table S4 for patient, treatment, and sample characteristics; STAR Methods). This study reported the appearance of novel expanded T cell clones, with an activated and exhausted phenotype and enhanced TCF7 expression following anti-PD-1 treatment for metastatic basal cell carcinoma. However, associations with clinical response were not investigated. We identified expanded TCRs present pre-treatment and tracked them post-treatment. We observed a trend for increased maintenance of expanded pre-existing clones in anti-PD-1 responders (p = 0.08) (Figure S8B), consistent with our findings in ADAPTeR. Taken together, these findings in two different indications suggest that anti-PD-1 is able to both expand novel T cell clones (likely driven by new T cell priming) and maintain previously expanded T cell clones, but only the latter appears to directly associate with clinical outcomes. Evaluation in larger datasets across tumor types with longitudinal tumor samples are needed to establish if TCR clonal maintenance is a universal feature of anti-PD-1 responders.

Antigen-specific T cell responses are often associated with the presence of clusters of TCRs with similar CDR3 peptide binding sequences (Dash et al., 2017; Glanville et al., 2017). We performed clonotype clustering analysis (STAR Methods) in the ADAPTeR cohort, and observed that expanded TCR clones showed a trend toward increased clustering of similar CDR3 sequences (or “cluster structure”) in responders compared with non-responders, both pre- and post-treatment (p = 0.06 and 0.07, respectively) (Figures 5F and S8C). At baseline, expanded TCRs that were maintained displayed significantly more cluster structure than expanded TCRs that were replaced (p = 0.008, Figures S8C–S8E). Taken together, these data suggest that in responders, there is a population of TCR clonotypes that have expanded in the tumor pre-treatment, and are preferentially maintained by anti-PD-1 treatment, perhaps reflecting enhanced stimulation by persistent antigen(s) and the ability of anti-PD-1 to prevent disappearance of such cells likely though prevention of programmed cell death (Wei et al., 2018). In non-responders, there was less TCR expansion pre-treatment and there was a more dynamic process of TCR replacement post-treatment, perhaps reflecting a lack of persistent antigen stimulation.

Finally, to investigate TCR repertoires across space and time, we performed TCR-seq on five disease sites in a patient enrolled in ADAPTeR who also underwent postmortem sampling (ADR005). This patient presented a mixed picture in that primary tumor and lung metastases maintained response to nivolumab until death; while new brain, bone, and thoraco-nodal metastases emerged on nivolumab, presenting sites of immune escape (Figure S9A). Five TCR clones were expanded pre-nivolumab in the primary tumor and lung metastasis and detected on-treatment (week 9). Following death, three of the five clones were maintained and expanded in non-progressive disease sites (primary tumor and lung), and none were detectable in the progressing sites (brain, bone, and thoraco-nodal metastases) (Figures S2D and S9B). Primary tumor, lung, and brain metastases were genetically similar, sharing 74% of all nsSNV/fsINDELs (Figures 1B and S9C). Of the 25 neoantigen-encoding mutations (55 predicted neoantigen-HLA binding pairs), eight were expressed across primary tumor, lung, and brain metastases (Figure S9B). Three neoantigen-encoding mutations (with five predicted neoantigen-HLA binding pairs) were exclusive to nivolumab-responsive sites, but relevance of this finding is unclear without direct confirmation of immune reactivity.

### Nivolumab binds pre-expanded CD8^+^ T cells and induces a cytotoxic phenotype in responders

To further characterize the CD8^+^ T cells exhibiting features of antigen engagement and potentially impacted by PD-1 blockade, we next sought to evaluate the transcriptional program of nivolumab-bound CD8^+^ T cells in samples obtained post therapeutic intervention. Due to large amounts of fresh tissue required for this analysis, it was only feasible in the two patients who underwent week 9 cytoreductive nephrectomy per study protocol. We derived and pooled single-cell suspensions of tumor-infiltrating lymphocytes from six spatially distinct regions of the nephrectomy specimens from ADR013 (responder) and ADR001 (non-responder) sorted nivolumab-bound CD8^+^ T cells and analyzed them via high-dimensional flow cytometry and single-cell RNA (scRNA-seq) and single-cell TCR (scTCR-seq) sequencing (STAR Methods). Detection of nivolumab (human immunoglobulin [Ig]G4) bound to cells with anti-IgG4 antibodies has previously been shown as a robust tool to evaluate PD-1 receptor occupancy by anti-PD-1 antibodies (Brahmer et al., 2010; Huang et al., 2017). We established the technical feasibility for detection of nivolumab-bound cells in a competition assay where IgG4 identified T cells bound to pembrolizumab (anti-PD-1 antibody) (Figure S10; STAR Methods) and applied this method to downstream assays in ADAPTeR.

Nivolumab-bound (IgG4^+^) CD8^+^ T cells showed higher expression of GZMB (38.9% versus 8.75%), TCF7 (19.5% and 2.17%), CD39 (54.6% versus 3.25%), TOX (14.5% versus 4.10%), and TIM3 (35.4% versus 3.52%) in ADR013 (responder) compared with ADR001 (non-responder) (Figure S11). This suggests that nivolumab-bound CD8^+^ T cells in the responder have a cytotoxic and progenitor-like phenotype (Ghorani et al., 2020; Miller et al., 2019; Wang et al., 2019; Kallies et al., 2020; Khan et al., 2019; Sekine et al., 2020; Yao et al., 2019; Zhao et al., 2021) and are likely tumor-reactive (Duhen et al., 2018; Simoni et al., 2018) (Table S5), despite upregulating markers of dysfunction. We also detected unbound PD-1 on nivolumab-bound CD8^+^ T cells in ADR013 (20.9%) and ADR001 (0.78%) (Figure S11), possibly indicating further PD-1 upregulation following nivolumab binding and TCR stimulation, i.e., as activation (Dong et al., 1999; Barber et al., 2006), particularly in the responder, rather than incomplete receptor occupancy.

**Figure 6:**
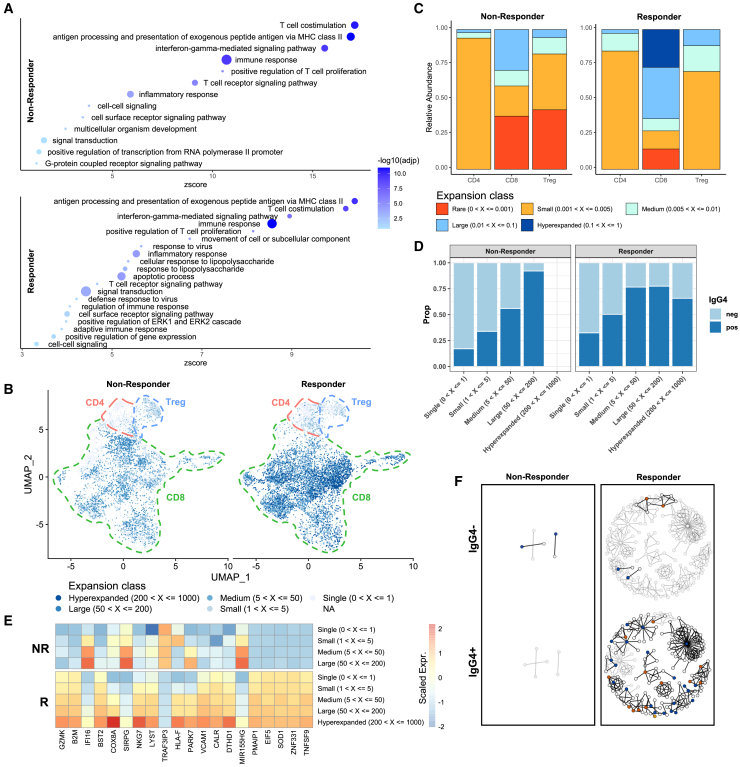
Nivolumab binding correlates with upregulation of T cell activation genes and clones expanded through persistent antigenic stimulation (A) GOBP pathway analysis of genes preferentially upregulated in drug-bound CD8^+^ cells in ADR001 (non-responder) and ADR013 (responder), circle size indicative of number of genes overlapping with GOBP term. (B) Uniform manifold approximation and projection (UMAP) of scRNA-seq data from non-responder and responder colored by frequency of clone. (C) Clonal proportion plot of CD8, CD4 effector, and Treg compartments in non-responder and responder. (D) Heatmaps showing top genes which positively correlated (Pearson’s correlation, CD8^+^ cells only) with TCR expansion in the responder. (E) Proportion of cells in each expansion class that are nivolumab-bound or unbound. (F) Representative network diagrams of post-treatment intratumoral CDR3 β-chain sequences for ADR001 and ADR013. Clustering was performed within the bulk TCR-seq data around expanded intratumoral TCRs, subdivided between clones that were expanded in the post-treatment repertoire exclusively (blue circles) and clones that were also expanded pre-treatment (orange circles). The network shows clusters for which at least one CDR3 was also detected in the scTCR repertoire. IgG4 negative clones that were detected in the scTCR repertoire but not expanded in the bulk TCR repertoire and are represented (yellow circle). The network was then split between clones that were mapping to a majority of IgG4 negative cells (top) or a majority of IgG4 positive cells (bottom) in the single-cell data. Clustering networks derived from bulk post-treatment tissue are shown (gray circles). See also Figures S10–S13 and Table S5.

Next, we performed paired single-cell RNA and TCR-seq (scRNA/TCR-seq), on the nivolumab-bound T cells (STAR Methods). scRNA-seq was annotated with the corresponding VDJ information for each cell and then merged. Cells were classed as CD8 (CD8^+^CD4^−^FOXP3^−^), CD4 (CD8^−^CD4^+^FOXP3^−^) and Tregs (CD8^−^FOXP3^+^) (Figure S12A; STAR Methods). We observed similar levels of CD8^+^ T cells, but lower proportions of Tregs in ADR013 (responder) compared with ADR001 (Figure S12B). Differential gene and gene set enrichment analyses of nivolumab-bound CD8^+^ T cells showed upregulated pro-inflammatory cyto/chemokine genes and T cell activation pathways in both cases (Figures 6A and S12C), irrespective of clinical response to nivolumab. We observed hyperexpanded CD8^+^ clones (defined as 200–1000 TCR clones with the same CDR3 sequence) in ADR013 but not in ADR001, where TCR expansion was limited (no expansion (singletons) to <200 clones) (Figures 6B and 6C). Further, expanded nivolumab-bound CD8^+^ T cells in ADR013 expressed higher levels of GZMK compared with ADR001 (Figures 6D and S12D). scRNA-seq data confirmed higher expression of GZMB, TCF7, TIM3, and CD39 expression in ADR013 on nivolumab-bound CD8^+^ T cells observed with flow cytometry (Figure S13). The proportion of nivolumab-bound CD8^+^ T cells was correlated with clonality (Figure 6E), suggesting that nivolumab binding leads to clonal expansion. T cell activation/dysfunction of nivolumab-bound cells, determined by publicly available gene signature of T cell states (STAR Methods) was evident in both patients, higher in ADR013, consistent with increased TCR stimulation of nivolumab-bound T cells in the responder (Figure S12E; STAR Methods).

Combining bulk and single-cell TCR-seq datasets, we evaluated if the expanded clones post-treatment (1) displayed cluster structure; (2) if clustered clones were drug-bound; and (3) if clustered, drug-bound clones were novel or pre-existing. We constructed cluster networks for ADR013 and ADR001 (STAR Methods), and defined each TCR clone within the networks by drug-binding status (IgG4^+^ or IgG4^−^). Then, we used pre/post-treatment bulk TCR-seq data to derive “novel” or “pre-existing” labels for each clone that was captured post-treatment by scTCR-seq (Figure 6F). In ADR013 (responder), expanded clones were clustered and mostly (89%) drug-bound, consisting of both pre-existing and novel TCRs (Figure 6F). By contrast, there was an overall paucity of expanded or clustered TCRs in ADR001 (non-responder), either novel or pre-existing (Figure 6F). This is consistent with the post-treatment bulk-level data in this patient and at cohort-level, where non-responders are characterized by clonal replacement of expanded TCRs. This limits inference on the relationship between clustering and drug-binding at the single-cell level in this non-responder patient.

While scRNA/TCR-seq data were derived from only two patients, they recapitulate the findings at the cohort-level data and provide further evidence for reinvigoration of pre-existing CD8^+^ T cells in responders. Critically, the data provide direct evidence that intratumoral T cells in a responding patient were expanded, PD-1 expressing, and nivolumab binding, and had a more activated phenotype, distinct from CD8^+^ T cells in the non-responder.

### Meta-analysis of >100,000 CD8^+^ T cells reveal expanded TCRs and GZMB/K upregulation in responders to CPI

**Figure 7:**
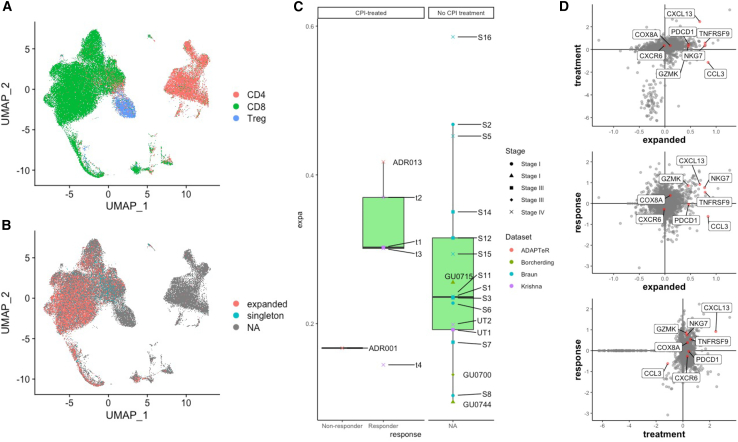
Meta-analysis of scRNA/TCR-seq data across Braun et al., Krishna et al., Borcherding et al., and ADAPTeR cohorts (A) Uniform manifold approximation and projection (UMAP) of merged CD8^+^ (CD8^+^/CD4^−^/FOXP3^−^), CD4^+^ effector (CD8^−^/CD4^+^/FOXP3^−^), and Treg (CD8^−^/FOXP3^+^) cells from four cohorts: Braun et al., Krishna et al., Borcherding et al., and ADAPTeR (ADR001 and ADR013), colored by cell types. (B) UMAP of scTCR-seq data of all cohorts colored by TCR expansions status (expanded or singleton clonotypes). Only CD8^+^ T cells are represented in color, NA denotes CD4^+^ T cells, Tregs, and unannotated CD8^+^ TCR clones (STAR Methods). (C) The TCR clonal expansion index is shown for each patient (median value of multiple regions for each patient where applicable). Patients are split between responders and non-responders of CPI treatment, or no CPI treatment. Disease stages (I–IV) are annotated. Only CD8^+^ T cell data are shown. Patient annotations from each cohort are: ADAPTeR – ADR013 (responder), ADR001 (non-responder); Brocherding et al. – GU0700, GU0744, GU0715; Braun et al. – S1, S2, S3, S5, S6, S7, S8, S11, S12, S14, S15, S16; Krishna et al. – t1, t2, t3, t4, UT1, UT2. Two-sided Mann-Whitney test p value shown; n = 23 patients. The minimum and maximum are indicated by the extreme points of the box plot; the median is indicated by the thick horizontal line; and the first and third quartiles are indicated by box edges. (D) Principal component analysis (PCA) analysis shows the differential gene expression pattern in expanded and non-expanded TCRs in CD8 cells based on CPI treatment and response status in the Braun et al., Krishna et al., Borcherding et al., and ADAPTeR cohorts. See also Figure S14.

Next, we sought to validate our findings in additional datasets. Three studies have reported ccRCC single-cell profiles across disease stages (Braun et al., 2021; Borcherding et al., 2021) or in the context of ipilimumab (anti-CTLA4) plus nivolumab (Krishna et al., 2021). Cohorts reported by Braun et al. (2021) (n = 12) and Borcherding et al. (2021) (n = 3) were treatment-naive patients, whereas Krishna et al. (2021) (n = 6) reported on patients treated with nivolumab (n = 1) or ipilimumab plus nivolumab (n = 3). We performed a meta-analysis of scRNA/TCR-seq data across these published studies (Braun et al., 2021; Krishna et al., 2021; Borcherding et al., 2021), as well as ADAPTeR, evaluating 45 tumor regions from 23 patients, totaling 159,688 cells after filtering for CD8/CD4/Treg cells (see Table S6 for patient, treatment, and sample characteristics; STAR Methods). As these samples were taken at single timepoints, longitudinal changes could not be assessed. To maximize comparability across cohorts, we applied a harmonized definition of CPI response (PFS >6 months on CPI classed as “responder”; PFS <6 months as “non-responder”) and TCR expansion (STAR Methods). In total, we collated scRNA profiles from 159,688 cells and TCR clonotypes from 21,053 cells, representing CD8 (n = 109,294), CD4 (n = 41,247) and Treg (n = 9,147) cells (Figures 7A, S14A, and S14B).

TCR clonal expansion was highly variable across disease stages (I-IV) but were typically grouped according to CPI response (higher in responders compared with the non-responder; p = 0.38) (Figures 7B and 7C). Among the responders, patient t4 was a clear outlier among the responders with a low degree of TCR clonal expansion, likely reflecting low CD8^+^ T cell (n = 1,631) and TCR capture (detected in 16% of cells) in these samples, compared with cohort median (3,856 CD8^+^ T cells and 59% TCR detection rate).

**Figure 8:**
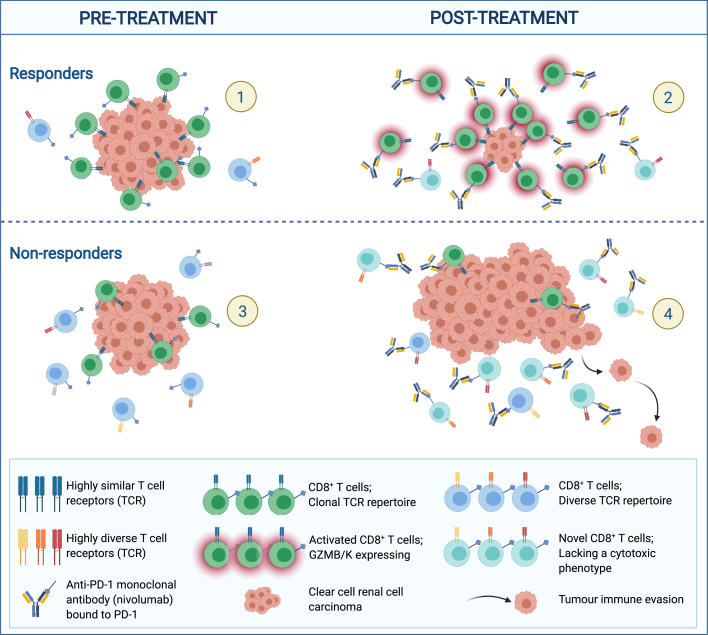
Longitudinal profiling by bulk and single-cell RNA/TCR-seq reveal dynamic immune correlates of response and resistance to nivolumab. (1) Clonally expanded CD8^+^ T cells pre-treatment in ADR013 (responder). High TCR clonality. (2) Maintenance of pre-existing clonally expanded and expansion of novel CD8^+^ T cells under nivolumab. Drug-binding activates CD8^+^ T cells during therapy response. (3) Limited clonal expansion of CD8^+^ T cells pre-treatment in non-responders. Low TCR clonality. (4) Replacement of expanded CD8^+^ T cells under nivolumab. Drug-binding occurs on CD8^+^ T cells that lack a cytotoxic phenotype and tumor progression ensues.

Evaluating all TCR clones, we observed higher expression of GZMB, PDCD1 (PD-1), HAVCR2 (TIM-3), and ENTPD1 (CD39) in CD8^+^ T cells from patients treated with CPI compared with untreated patients (Figure S14A), and in CPI-responders compared with non-responders (Figure S14B). Expanded TCR clones had higher expression of activation (i.e., GZMB, IFNG, HLA-DR, CCL3) and immune checkpoint markers (i.e., HAVCR2, LAG3, CTLA4) (Figure S14C). Expanded TCRs in responders but not the non-responder showed upregulation of CD137 (TNSFR9, 4-1BB), a co-stimulatory molecule that interacts with antigen-presenting cells to support T cell anti-tumor activity (Ye et al., 2014; Thommen and Schumacher, 2018) and express GZMK (Figure 7D). Despite inherent differences in timing of sampling and CPI regimens across these cohorts, the data are consistent with the preferentially expansion of activated/exhausted CD8^+^ T cells in responders to PD-1 blockade (Figure 8).

## Discussion

We present results of a phase II study and multi-omic analysis of advanced stage ccRCC through treatment that sheds light on the determinants of anti-PD-1 response and resistance, and in particular the nature of the CD8^+^ T cells likely contributing to anti-tumor immunity.

No single mutation, SCNA, nor TMB and INDEL load associated with response in accordance with prior studies (Braun et al., 2020; McDermott et al., 2018; Motzer et al., 2020b), although our small cohort size was likely underpowered to robustly detect response associations with genomic alterations. The question about the contribution of mutations or SCNA events to anti-tumor immunity in ccRCC remains incompletely understood. A notable exception was a case with excessively high TMB mediated by MMRD, and *B2M* was likely selected to provide immune escape after these sites acquired MMRD/high neoantigen load. Decreased MHC-I expression associates with reduced PFS with avelumab (anti-PD-L1) plus axitinib (anti-vascular endothelial growth factor) in ccRCC (Motzer et al., 2020b), but the frequency and impact of loss of antigen presentation is undefined. The role of mutations in forming neoantigens in ccRCC remains unclear, but we note reports of T cell reactivities to mutant peptides both from point mutations and INDELs (Rahma et al., 2010). The difficulty in linking mutations, especially fsINDELs, to response may lie in the accuracy of variant calling, and overall low response rate to anti-PD-1 monotherapy.

We show that HERVs frequently associated with T cell infiltration in bulk tumor biopsies (Rooney et al., 2015; Panda et al., 2018; Smith et al., 2018; Takahashi et al., 2008), such as ERV3-2 and ERVK-10, are highly expressed in immune cells. This offers a more parsimonious explanation for previously described associations to both T cell infiltration and CPI response. We found previous HERV nomination methods were problematic (fragmented, incomplete, or multi-gene spanning transcripts) and this is an additional barrier to HERV signatures translating to a clinical predictive biomarker. We confirmed that ERVE-4 and HERV4700 are ccRCC-specific, consistent with studies demonstrating direct T cell reactivity to these specific HERVs (Takahashi et al., 2008). While they did not associate with nivolumab response in this cohort, we note that T cell responses targeting these HERVs are HLA-A^∗^02 and HLA-A^∗^11 restricted (Smith et al., 2018; Cherkasova et al., 2016), and consequently, a positive correlation with the outcome of immunotherapy would only be expected in patients with this HLA allele. Overall, these findings have implications for approaches in ongoing CPI-biomarker discovery and potential therapeutic targeting of HERVs in ccRCC.

While the source of antigenic stimulus in ccRCC remains elusive, antigen-agnostic evaluation of TCR repertoire offered new and relevant insight into the impact of anti-PD-1 on T cell responses. Among these, the existence of a tumor-specific T cell response is supported by our findings of pre-existing, expanded CD8^+^ T cell clones in responders, and the maintenance of these expanded CD8^+^ T cell clones characterizes response to nivolumab. These findings, which were directly demonstrated using our longitudinal samples, validate the previous report of expanded tissue-resident T cells in a CPI responder inferred from pseudotime analysis of post-treatment single-cell data (Krishna et al., 2021). Moreover, while previous studies have demonstrated that TCR clonality varies considerably across disease stages (Braun et al., 2021), our data identify baseline pre-treatment TCR clonality as a predictor of a clinical response to anti-PD-1. This observation of a potential biomarker may be important in the adjuvant setting, given phase III clinical trial data (KEYNOTE-564; NCT03142334) showing improved disease-free survival in patients with high-risk resected ccRCC receiving pembrolizumab (Choueiri et al., 2021).

We show that on-treatment change in GZMB expression is a dynamic biomarker of nivolumab in ccRCC, and increase in TCF7^+^CD8^+^ T cells and B cells also correlated with response in our cohort. While we acknowledge that these findings would benefit from validation with larger longitudinal datasets, GZMB has also demonstrated predictive utility for neoadjuvant avelumab in bladder cancer (Powles et al., 2019), and a prior report in ccRCC has shown TCF7^+^CD8^+^ T cell can be activated *in vitro*, and could maintain a progenitor-like state when located within antigen presentation niches (Jansen et al., 2019). Higher CD8^+^ T cell density at tumor invasive margin has been reported to associate with longer PFS with avelumab plus axitinib in ccRCC (Motzer et al., 2020b). As such, further work to characterize the interaction between co-located B and T cells, especially at tumor margins, will be critical.

There are limitations to our study. First, the small number of patients limit data generalizability, and findings from this study would benefit from validation in larger datasets; however, our scope for discovery was afforded by a broadened sampling frame (multiregion and multi-metastatic site biopsies) and longitudinally tracking of molecular and tumor immune microenvironment (TIME) changes under therapy. Samples from only two patients underwent multiparameter flow cytometry and scRNA/TCR-seq analyses in our study, and results remain exploratory. While this facilitated high-resolution cellular characterization, spatial relationship with other immune cells was not evaluable. Looking forward, spatial transcriptomic profiling techniques with single-cell sensitivity (Merritt et al., 2020; Rodriques et al., 2019) will be valuable in studying TIME evolution in ccRCC. Finally, while results from the meta-analysis support findings in ADAPTeR, cross-study differences in cohort and treatment characteristics remain a key consideration to interpretation of these results, including the possibility that the addition of ipilimumab in the Krishna et al. (2021) cohort may confound observed immune responses.

In conclusion, in this prospective study, we reveal features of anti-PD-1 response and resistance in ccRCC. We identified tumor-specific T cells with cytotoxic features in ccRCC, which hold promise for development of adoptive cellular therapy for this cancer (Wong et al., 2017). While the treatment landscape has evolved to include combination therapies (Albiges et al., 2019), this dissection of immune changes under nivolumab provides the foundation for understanding response to combination therapies, and is relevant to the application of anti-PD-1 in the adjuvant setting (Choueiri et al., 2021). Finally, our multi-omic analysis framework provides a template and highlights challenges for future immuno-oncology biomarker studies in ccRCC.

## STAR★Methods

### Key resources table

**Table:** 

REAGENT or RESOURCE	SOURCE	IDENTIFIER
**Antibodies**		

Mouse anti-human monoclonal anti-CD8 [RPA-T8; BUV496]	BD Biosciences	Cat#612942; RRID: AB_2870223
Mouse anti-human monoclonal anti-CD45RA [HI100; BUV563]	BD Biosciences	Cat#612926; RRID: AB_2870211
Mouse anti-human monoclonal anti-CD4 [SK3; BUV615]	BD Biosciences	Cat#612987; RRID: AB_2870258
Mouse anti-human monoclonal anti-CD38 [HIT2; BUV737]	BD Biosciences	Cat#741837; RRID: AB_2871172
Mouse anti-human monoclonal anti-CD3 [SK7; BUV805]	BD Biosciences	Cat#612893; RRID: AB_2870181
Mouse anti-human monoclonal anti-FOXP3 [206D; BV421]	BioLegend	Cat# 320124; RRID: AB_2565972
Mouse anti-human monoclonal anti-CD194 (CCR4) [L291H4; BV510]	BioLegend	Cat# 359415; RRID:AB_2562436
Mouse anti-human monoclonal anti-CD57 [QA17A04; BV605]	BioLegend	Cat#393304; RRID AB_2728426
Mouse anti-mouse/human monoclonal anti-Ki-67 [B56; BV650]	BD Biosciences	Cat# 563757; RRID AB_2688008
Mouse anti-human monoclonal anti-CD39 [TU66; BV711]	BD Biosciences	Cat# 563680; RRID AB_2738369
Mouse anti-human monoclonal anti-CD197 (CCR7) [G043H7; BV750]	BioLegend	Cat#353254; RRID AB_2800945
Mouse anti-human monoclonal anti-CD69 [FN50; BV786]	BioLegend	Cat#310932; RRID AB_2563696
Mouse anti-human monoclonal anti-CD103 [Ber-ACT8; BB515]	BD Biosciences	Cat#564578; RRID AB_2738852
Mouse anti-human monoclonal anti-CD185 (CXCR5) [J252D4; PerCp-Cy5.5]	BioLegend	Cat# 356909; RRID AB_2561818
Mouse anti-human monoclonal anti-TCF1 (TCF7) [7F11A10; PE]	BioLegend	Cat#655208; RRID AB_2728492
Mouse anti-human monoclonal anti-Granzyme B [GB11; PE-CF594]	BD Biosciences	Cat#562462; RRID AB_2737618
Mouse anti-human monoclonal anti-CD25 [M-A251; PE-Cy5]	BD Biosciences	Cat#555433; RRID AB_395827
Mouse anti-human monoclonal anti-CD279 (PD-1) [EH12.2H7; PE-CY7]	BioLegend	Cat#329918; RRID AB_2159324
Mouse anti-mouse/human monoclonal anti-TOX [REA473; APC]	Miltenyi Biotec	Cat#130-118-335; RRID: AB_2751485
Mouse anti-human monoclonal anti-IgG4 [Biotin]	Invitrogen	Cat# MH1542; RRID: AB_2539712
Mouse anti-human monoclonal anti-CD137 (4-1BB) [4B4-1; BUV661)	BD Biosciences	Cat#741642; RRID AB_2871042
Mouse anti-human monoclonal anti-TIM-3 (CD3660 [7D3; BV650]	BD Biosciences	Cat#565564; RRID AB_2722547
Mouse anti-human monoclonal anti-CD138 [MI15]	BD Biosciences	Cat#551902; RRID AB_394291
Mouse anti-human monoclonal anti-*MLH1* [M1]	Sigma-Aldrich (Merck)	Cat#WH0004292M2; RRID: AB_1842488
Rabbit anti-mouse/human monoclonal anti-beta-2 microglobulin [4H5L6]	ThermoFisher Scientific	Cat#701250; RRID AB_2532441
Live/dead fixable yellow cell death stain [BV570]	ThermoFisher	Cat#L34968; RRID N/A
Streptavidin [BUV395]	BD Biosciences	Cat#564176; RRID AB_2869553
Mouse anti-human monoclonal anti-CD3 [LN10]	Leica	Cat#CD3-565-L-CE; RRID AB_563541
Mouse anti-human monoclonal anti-CD4 [4B12]	Leica	Cat#CD4-368-L-CE; RRID N/A
Mouse anti-human monoclonal anti-CD8 [4B11]	Leica	Cat#CD8-4B11-L-CE; RRID AB_10555292
Mouse anti-human monoclonal anti-Granzyme B [11F1]	Leica	Cat#GRAN-B-L-CE; RRID N/A
Mouse anti-human monoclonal anti-FOXP3 [236A/E7]	Abcam	Cat#ab20034; RRID AB_445284
Mouse anti-human monoclonal anti-CD163 [10D6]	Leica	Cat#CD163-L-CE; RRID N/A
Rabbit anti-human monoclonal anti-CD19 [SP291]	Spring Bioscience	Cat#M5914; RRID N/A
Mouse anti-human monoclonal anti-CD138 [MI15]	Agilent Dako	Cat#M7228; RRID AB_2254116
Mouse anti-human monoclonal anti-PD-1 [NAT105]	Abcam	Cat#ab52587; RRID AB_881954

**Deposited data**		

Multi-region whole-exome sequencing data on ADAPTeR patient cohort.	This study	EGAS00001005638
Multi-region RNA-seq data on ADAPTeR patient cohort.	This study	EGAD00001008163
Multi-region TCR-seq data on ADAPTeR patient cohort.	This study	EGAD00001008165
Multi-region single-cell RNA and TCR-seq data on ADAPTeR patients.	This study	EGAD00001008166
Multi-region single-cell RNA and TCR-seq single-cell count matrices, VDJ annotations, and metadata on ADAPTeR patients	This study	https://doi.org/10.5522/04/16573640.v1
Multi-region processed bulk-TCR sequence data	This study	https://doi.org/10.5522/04/16571573.v1
Next generation sequencing of human immune cell subsets across diseases	Linsley et al., 2014	GSE60424
RNA-seq of human AML, CMML and MDS CD34+ blast cells, CD4+ T cells and CD8+ T cells treated with 5-aza against untreated samples and healthy controls	Kazachenka et al., 2019	E-MTAB-8208
Yost et al. cohort TCRseq data derived from tumours samples from patients with metastatic basal cell carcinoma pre- and post-anti-PD-1 treatment	Yost et al., 2019	https://doi.org/10.21417/KY2019NM; https://clients.adaptivebiotech.com/pub/yost-2019-natmed
Braun et al. cohort scRNA/TCRseq data derived from tumour samples from patients with stage I-IV ccRCC	Braun et al., 2021	Downloaded from the published supplemental data
Borcherding et al. cohort scRNA/TCRseq data derived from tumour samples from patients with stage I-III ccRCC	Borcherding et al., 2021	GSE121638
Krishna et al. cohort scRNA/TCRseq data derived from tumour samples from patients with stage III & IV ccRCC	Krishna et al., 2021	https://trace.ncbi.nlm.nih.gov/Traces/sra/sra.cgi?analysis=SRZ190804

**Oligonucleotides**		

Oligonucleotide sequences for *VHL* methylation-specific PCR see STAR Methods	This study	N/A
Oligonucleotide sequences for *B2M* specific PCR to detect mutations by Sanger sequencing see STAR Methods	This study	N/A

**Software and algorithms**		

Burrows-Wheeler Aligner (BWA) v0.7.15	Li and Durbin, 2009	http://bio-bwa.sourceforge.net/
Samtools v1.3.1	Li and Durbin, 2009	http://samtools.sourceforge.net/
Picard v1.81	N/A	http://broadinstitute.github.io/picard/
Mutect v1.1.7	Cibulskis et al., 2013	http://archive.broadinstitute.org/cancer/cga/mutect
VarScan v2.4.1	Koboldt et al., 2009	http://varscan.sourceforge.net/
Scalpel v0.5.3	Fang et al., 2016a	https://github.com/hanfang/scalpel-protocol
Annovar	Wang et al., 2010a	http://annovar.openbioinformatics.org/en/latest/
Polysolver v1.0.0	Shukla et al., 2015	https://software.broadinstitute.org/cancer/cga/polysolver
NetMHCpan v3.0	Andreatta and Nielsen, 2016	http://www.cbs.dtu.dk/services/NetMHCpan-3.0/
NetMHC v4.0	Andreatta and Nielsen, 2016	http://www.cbs.dtu.dk/services/NetMHC/
Platypus v0.8.1	Rimmer et al., 2014	https://github.com/andyrimmer/Platypus
CNVkit v0.7.3	Talevich et al., 2016	https://github.com/et al./cnvkit
mapsplice v2.2.0	Wang et al., 2010b	http://www.netlab.uky.edu/p/bioinfo/MapSplice2
R package PSCBS v0.61.0	Olshen et al., 2011	https://cran.r-project.org/web/packages/PSCBS/index.html
R package deconstructSigs v1.8.0	Rosenthal et al., 2016	https://github.com/raerose01/deconstructSigs
R package Copynumber v1.12.0	Nilsen et al., 2012	http://bioconductor.org/packages/release/bioc/html/copynumber.html
R package ABSOLUTE v1.2	Carter et al., 2012	http://archive.broadinstitute.org/cancer/cga/absolute
R package Rsamtools v1.3.1	Morgan et al., 2017	https://bioconductor.org/packages/release/bioc/html/Rsamtools.html
bedtools package	Quinlan and Hall, 2010	http://bedtools.readthedocs.io/en/latest/
STAR aligner v2.6.1	Dobin et al., 2013	https://github.com/alexdobin/STAR
RSEM v1.3.0	Li and Dewey., 2011	https://github.com/deweylab/RSEM
Hisat2 v.2.1.0	Kim et al., 2019	http://daehwankimlab.github.io/hisat2/
Subread package v.1.5.0	Liao et al., 2014	http://subread.sourceforge.net/
Lift Genome Annotations Tool	N/A	https://genome.ucsc.edu/cgi-bin/hgLiftOver
BLASTn	Camacho et al., 2009	https://blast.ncbi.nlm.nih.gov/Blast.cgi?PROGRAM=blastn&BLAST_SPEC=GeoBlast&PAGE_TYPE=BlastSearch
DESeq2	Love et al., 2014	https://bioconductor.org/packages/release/bioc/html/DESeq2.html
R package ‘XGR’	Fang et al., 2016b	https://xgr.r-forge.r-project.org/
innate2adaptive/Decombinator	N/A	https://github.com/innate2adaptive/Decombinator
R package ‘kernlab’	Karatzoglou et al., 2004	https://rdrr.io/cran/kernlab/
10x Genomics Cell Ranger 5.0.0	Zheng et al., 2017	https://support.10xgenomics.com/single-cell-gene-expression/software/overview/welcome
Seurat v.4.0.3	Stuart et al., 2019	https://satijalab.org/seurat/
SCTransform	Hafemeister and Satija, 2019	https://github.com/ChristophH/sctransform
Harmony	Korsunsky et al., 2019	https://portals.broadinstitute.org/harmony/
MAST	Finak et al., 2015	https://www.bioconductor.org/packages/release/bioc/html/MAST.html
scRepertoire	Borcherding et al., 2020	https://github.com/ncborcherding/scRepertoire
STARTRAC	Zhang et al., 2018b	https://github.com/Japrin/STARTRAC

**Other**		

ADAPTeR: A Study of Anti-PD1 (Nivolumab) Therapy as Pre- and Post-operative Therapy in Metastatic Renal Cell Cancer	This study	NCT02446860

### Resource availability

#### Lead contact

Further information and requests for resources and reagents should be directed to and will be fulfilled by the Lead Contact, Samra Turajlic (samra.turajlic@crick.ac.uk).

#### Materials availability

This study did not generate new unique reagents.

### Experimental model and subject details

#### Clinical studies

ADAPTeR (NCT02446860) is a single-arm, open-label, phase II study of nivolumab therapy as pre-operative therapy in metastatic clear cell renal cell carcinoma (ccRCC). Planned interim analysis took place after six months after the last patient enrolled had their first Response Evaluation Criteria in Solid Tumours (RECIST version 1.1) defined objective response assessment. ADAPTeR was initially approved by NRES Committee London Fulham on 01/12/2014. ADAPTeR is performed in accordance with the ethical principles in the Declaration of Helsinki, Good Clinical Practice and applicable regulatory requirements.

Nivolumab was administered at a dose of 3mg per kilogram of body weight as a 60 minute intravenous infusion every 2 weeks. Eligible patients were 18 years of age or older, had histologic confirmation of advanced or metastatic ccRCC with predominantly clear cell component with at least one site of disease outside the kidney measurable according to the RECIST version 1.1, with no prior systemic therapy for ccRCC. All patients had an Eastern Cooperative Oncology Group (ECOG) performance status of 0 or 1. Key exclusion criteria were need for immediate nephrectomy, any active, known or suspected autoimmune disease or another condition requiring systemic treatment with either corticosteroids (>10mg daily prednisolone equivalent) or other immunosuppressive medications within 14 days of study drug administration (excluding vitiligo, Type 1 diabetes mellitus, residual hypothyroidism due to autoimmune condition only requiring hormone replacement, psoriasis not requiring systemic treatment or conditions not expected to recur in the absence of an external trigger). During the course of the study, inclusion expanded to those who have had a prior nephrectomy but are suitable for on treatment biopsies. The prognostic factors assessed for the risk categorisation are as per the published IMDC criteria (Heng et al., 2009): time to systemic therapy (<1 year), performance status, anaemia, hypercalcaemia, neutrophilia and thrombocytosis. Presence of zero (favourable-risk), one (intermediate-risk), and two or three (poor-risk) factors provides the categorisation.

The primary endpoint was the safety profile of nivolumab given pre- and post-operatively to patients with metastatic ccRCC undergoing nephrectomy. Secondary endpoints were overall response rate (ORR), progression free survival (PFS), and overall survival (OS). Exploratory endpoints pertain to biomarker analyses. Patients deemed clinically suitable for nephrectomy at baseline were scheduled for surgery after the fourth cycle of treatment. Patients not deemed clinically suitable for nephrectomy at baseline would undergo surgery if an excellent clinical response is observed and if surgery was clinically appropriate. Nivolumab treatment was recommenced post-operatively upon sufficient recovery, and until disease progression. Patients who remained clinically unsuitable for nephrectomy continued nivolumab treatment until disease progression.

For translational study sample collection, baseline tumour biopsy via appropriate guidance (ultrasound or computer tomography [CT]) at least 3 days and up to 14 days prior to starting nivolumab was obtained. Tumour multiple regions of nephrectomy specimen were sampled, as well as image guided biopsy of regressing lesions or at disease progression either at site of progression or, if not possible, percutaneous primary renal tumour biopsy, prior to commencement of any subsequent treatment. Blood samples were collected at each tumour sampling timepoint.

Autopsy samples from ADR001, ADR005, and ADR015 were obtained through the PEACE Study (NIHR 18422; NCT03004755), where samples were harvested within 48 hours from death for these patients. All patients were co-recruited to the TRACERx Renal study (NCT03226886; see secondary author list for the full list of TRACERx Renal consortium investigators). Patient and sample metadata (i.e. age a diagnosis, sex, clinical response, biopsy site) are provided as Tables S1 and S2. All the patients provided written informed consent. The protocols, amendments and informed consent forms were approved by the institutional review board or independent ethics committee at each trial site for each trial.

### Method details

#### Sample collection

Tumour and normal tissue were collected via image-guided percutaneous biopsies, *ex vivo* sampling at nephrectomy, and at autopsy. Multi-region samples were obtained with all modalities. For samples obtained at nephrectomy, resected specimens were reviewed macroscopically by a pathologist to guide multi-region sampling for this study and to avoid compromising diagnostic requirements. Spatially separated regions sampled from the ‘‘tumour slice’’ using a 6mm punch biopsy needle. The punch was changed between samples to avoid contamination. The total number of samples obtained reflects the tumour size with a minimum of three biopsies that are non-overlapping and equally spaced. Areas which are obviously fibrotic or haemorrhagic are avoided during sampling and every attempt is made to reflect macroscopically heterogeneous tumour areas. Primary tumour regions are labelled as R1, R2, R3.Rn and locations are recorded. Normal kidney tissue was sampled from areas distant to the primary tumour and labelled N1. For all samples collected, each were split into two for snap freezing and formalin fixing respectively, such that the fresh frozen sample has its mirror image in the formalin-fixed sample which is subsequently paraffin embedded. Fresh samples were placed in a 1.8 ml cryotube and immediately snap frozen in liquid nitrogen for >30 seconds and transferred to -80 C for storage. Peripheral blood was collected at the time of surgery and processed to separate buffy coat and peripheral blood mononuclear cells (PBMCs).

#### Nucleic acid extraction, DNA and RNA library preparation and sequencing

DNA and RNA were co-extracted from fresh-frozen tumour tissue using AllPrep DNA/RNA mini kit (Qiagen). RNA from peripheral blood mononuclear cells (PBMC) were extracted from blood stored in Tempus tubes using the Tempus™ Spin RNA Isolation Kit (Invitrogen). Germline DNA was isolated from whole blood using the DNeasy Blood and Tissue kit (Qiagen). DNA yield and quality were assessed on TapeStation4200 (Agilent) and Qubit Fluorometric quantification (ThermoFisher Scientific). Samples were normalised to either 3 ug or 200ng and sheared to 150-200bp using a Covaris-E220 or LE220-plus. Agilent SureSelectXT enriched libraries were constructed following the manufacturer’s manual or automated (using the Agilent Bravo liquid handling platform) SureSelectXT Target Enrichment System for Illumina Paired-end Multiplexed Sequencing Library protocol. Hybridisation and capture were performed using the Agilent SureSelectXT Human All Exon v5 capture library. Final libraries were sequenced to a target coverage of 250x with 101bp paired-end reads multiplexed on the Illumina HiSeq4000 sequencing platform. The extracted RNA was normalised to 100ng for library construction using RNA-Ribozero (ribodeplete) Library Preparation Kits. The prepared libraries were multiplexed and QC’ed before paired-end sequencing with target coverage of 50 million reads per sample on HiSeq4000 sequencing platforms (Illumina). RNA was extracted from blood for TCR sequencing from the following cases and timepoints: all cases (n = 15) pre- and post-treatment.

#### SNV, and INDEL calling from multiregion WE sequencing

Paired-end reads (2×33100bp) in FastQ format sequenced by Hiseq were aligned to the reference human genome (build hg19), using the Burrows-Wheeler Aligner (BWA) v0.7.15. with seed recurrences (-c flag) set to 10000(Li and Durbin, 2009). Intermediate processing of Sam/Bam files was performed using Samtools v1.3.1 and deduplication was performed using Picard 1.81 (http://broadinstitute.github.io/picard/). Single Nucleotide Variant (SNV) calling was performed using Mutect v1.1.7 and small scale insertion-and-deletions (INDELs) were called running VarScan v2.4.1 in somatic mode with a minimum variant frequency (--min-var-freq) of 0.005, a tumour purity estimate (--tumour-purity) of 0.75 and then validated using Scalpel v0.5.3 (scalpel-discovery in --somatic mode) (intersection between two callers taken) (Fang et al., 2016a; Cibulskis et al., 2013; Koboldt et al., 2009). SNVs called by Mutect were further filtered using the following criteria: i) ≤5 alternative reads supporting the variant and variant allele frequency (VAF) of ≤1% in the corresponding germline sample, ii) variants falling into mitochondrial chromosome, HLA genes or any intergenic region were not considered, iii) presence of both forward and reverse strand reads supporting the variant, iv) >5 reads supporting the variant in at least one sample, v) variants were required to have a VAF of 0.01 in at least one sample, vi) sequencing depth need to be ≥20 and ≤3000 across all samples. Dinucleotide substitutions (DNV) were identified when two adjacent SNVs were called and their VAFs were consistently balanced (based on proportion test, *P* ≥ 0.05). In such cases the start and stop positions were corrected to represent a DNV and frequency related values were recalculated to represent the mean of the SNVs. Variants were annotated using Annovar (Wang et al., 2010a). Individual tumour biopsy regions were judged to have failed quality control and excluded from analysis based on the following criteria: i) sequencing coverage depth below 100×, ii) low tumour purity such that copy number calling failed. Driver variants are manually reviewed and predicted for variant effect and variant annotations on the heatmap are only for confident driver events.

#### Methylation specific PCR

Methylation of the *VHL* promoter was detected after bisulphite treatment of 500ng of patient DNA using the EZ DNA Methylation-Direct kit (Zymo Research). Bisulphite treated DNA was amplified in the PCR using methylation specific oligonucleotides followed by Big Dye terminator Sanger sequencing. Methylation was confirmed by comparing and contrasting patient tumour and normal renal tissue for methylation protected CpG sequences. Oligonucleotide names and sequences 5′-3’: VHL_methylation_1F (forward): gagtttttttaggttattttttgtaat; VHL_methylation_1R (reverse): tcaccctaaatatatatcctacctcaaaa; VHL_methylation_2F: cccctctaaaatttaatattttt; VHL_methylation_2R: ggttaaggttgtagtgagttaagtt.

#### Neoantigen calling

Neoantigen predictions were derived by first determining the 4-digit HLA type for each patient, along with mutations in class I HLA genes, using POLYSOLVER (Shukla et al., 2015). Next, all possible 9, 10 and 11-mer mutant peptides were computed, based on the detected somatic non-synonymous SNV and INDEL mutations in each sample. Binding affinities of mutant and corresponding wildtype peptides, relevant to the corresponding POLYSOLVER-inferred HLA alleles, were predicted using NetMHCpan (v3.0) (Hoof et al., 2008) and NetMHC (v4.0) (Andreatta and Nielsen, 2016). Neoantigen binders were defined as strong binders if their %rank was below <0.5 for the mutant and >0.5 for the wildtype protein.

#### TMB, fsINDEL burden, neonatigen burden, wGII, ITH index

Tumour mutational burden (TMB) was calculated as the number of exonic non-synonymous SNVs per mega base. The frameshift INDEL (fsINDEL) burden was calculated as the total number of exonic frameshift INDELs per sample. Clonal TMB/fsINDEL burden was accordingly calculated as the number of ubiquitous non-synonymous SNVs/fsINDELs (shared by all samples) in multi-region sampled cases and as the number of mutations with a CCF >0.5 for patients with single-region sampling. The neoantigen burden was calculated as the total number of predicted strong binders per sample. The average proportion of the genome with aberrant copy number, weighted on each of the 22 autosomal chromosomes, was estimated as the weighted genome instability index (wGII). Maximum wGII for each patient (from multiregion sample sets) was used as overall tumour wGII. Overall ITH was measured as an index (ITH index = # subclonal drivers/# clonal drivers, where ‘‘drivers’’ include all driver mutations and driver SCNAs shown in Figure 1B).

#### SNP calling

Single nucleotide polymorphisms (SNPs) were called in the germline sample using Platypus v0.8.1 with default parameters apart from --genIndels = 0 and --minMapQual = 40. Tumour regions were genotyped at positions where a SNP was detected in the germline (parameters set to --minPosterior = 0 --getVariantsFromBAMs = 0). SNPs with a minimum coverage of 50× in the germline and the tumour sample were used for allele-specific copy number segmentation.

#### Copy number analysis

CNVkit v0.7.3 was used with default parameters on paired tumour-normal sequencing data (Talevich et al., 2016). Outliers of the derived log2-ratio (logR) calls from CNVkit were detected and modified using Median Absolute Deviation Winsorization before case-specific joint segmentation of fresh-frozen samples to identify genomic segments of constant logR (Nilsen et al., 2012). Formalin-fixed and paraffin-embedded (FFPE) samples were segmented separately while leveraging the segment information from the fresh-frozen samples. Copy number alterations were called as losses or gains relative to overall sample wide estimated ploidy. Driver copy number was identified by overlapping the called somatic copy number segments with putative driver copy number regions identified by Beroukhim et al. (Beroukhim et al., 2009). Allele-specific segmentation was performed using the paired PSCBS method after removal of single-locus outliers (R package PSCBS v0.61.0) (Olshen et al., 2011).

#### Purity and ploidy estimate

Tumour sample purity, average ploidy and absolute allelic copy number per segment were estimated using ABSOLUTE v1.2 in allelic mode (Carter et al., 2012). In line with recommended best practice all ABSOLUTE solutions were reviewed by 3 researchers, with solutions selected based on majority vote. Purity assigned 0.1 for samples below ABSOLUTE estimate thresholds for comparison analysis of samples between responders and non-responders.

#### Subclonal deconstruction

To estimate the CCF of a mutation, we used the following formula:

$$VAF=\frac{CN_{mut}*CCF*p}{CN_{n}*\left(1-p\right)+CN_{t}*p}$$

Where VAF is the variant allele frequency of the mutation, p the estimated tumour purity, CN_mut_ the number of copies carrying the mutation and CN_t_ the local copy number in the tumour cells. CN_n_ is the local copy number in the non-tumour proportion of the sample which was assumed to be 2. The CN_mut_ and CCF were estimated through iteration of all possible combinations of CCF (range 0.01 to 1, by 0.01) and CN_mut_ (range 1 to CN_t_, by 1) using the formula above to identify the best fit CCF.

#### Selection against neoantigen-encoding mutations

For each patient with matched pre- and post-treatment WES data (N = 8 patients), the CCFs of all nsSNVs and fsINDELs were compared pre- and post-treatment. In patients with multiple pre-treatment samples, median pre-treatment CCFs were used as baseline. A mutation was defined to have undergone mutation depletion (‘genomic contraction’) (Riaz et al., 2017) if the CCF decreased by ≥ 10% from pre- to post-treatment or if the mutation was present in the pre-treatment but not the post-treatment sample. An enrichment test (Fisher’s exact test) was performed to determine whether mutations which are predicted to encode neoantigens were more likely to undergo genomic contraction than the remaining nonsynonymous SNVs and frameshift INDELs.

#### Mutational signature analysis

Mutational signatures were estimated using the deconstructSigs package in R (Rosenthal et al., 2016). Sample specific mutational signature analysis was restricted to samples with at least 50 mutations.

#### Analysis for mismatch repair deficiency

Analysis for mutations in the following nominated genes was performed: *POLD3, MLH3, MSH6, RPA4, LIG1, MLH1, MSH2, MSH3, PCNA, PMS2, POLD1, POLD2, POLD4, RFC1, RFC2, RFC3, RFC4, RFC5, RPA1, RPA2, RPA3, SSBP1, EXO1.*

#### Analysis for mutations associated with defective antigen presentation

Analysis for mutations in the following nominated genes was performed: *B2M, CIITA, IRF1, PSME1, PSME2, PSME3, ERAP1, ERAP2, HSPA, PSMA7, HSPC, HSPBP1, TAP1, TAP2, TAPBP, CALR, CNX, CANX, PDIA3*.

#### Detection of *B2M* mutations by Sanger sequencing

Validation of the *B2M* mutation was performed using PCR followed by Big Dye Terminator Sanger sequencing on the ABI 3700. 20ng of patient DNA was amplified for exon 1 of *B2M*, to enable detection of *B2M*:c.42_45delTCTT:p.S14fs. PCR conditions involved 35 cycles of denaturation at 950C, followed by oligonucleotide primer annealing at 55°C and sequence extension at 720C using Qiagen Taq polymerase and reagents. Oligonucleotide sequences used are: Forward: aacgggaaagtccctctctc; Reverse: agatccagccctggactagc.

#### Bulk RNAseq data processing

RNAseq data were mapped to the hg19 reference human genome using the STAR (Dobin et al., 2013) algorithm, and transcript and gene abundance were estimated by RSEM (Dobin et al., 2013) with default parameters. Samples were excluded if they had fewer than 15,000 genes detected.

#### Whole-transcriptome sequencing (RNA) variant calling

Insertion/deletion mutations were called from raw paired end FASTQ files, using mapsplice (v2.2.0), with sequence reads aligned to hg19 genomic assembly (using bowtie pre-built index). Minimum QC thresholds were set to retain variants with ≥5 alternative reads, and variant allele frequency ≥0.05. Insertions and deletions which were detected in both RNA and DNA sequencing assays for the same sample were designated as expressed indels. SNVs in RNA sequencing data were called directly from the BAM files, using Rsamtools to extract read counts per allele for each genomic position where a SNV had already been called in DNA sequencing analysis. Similarly, minimum QC thresholds of ≥5 alternative reads, and variant allele frequency ≥0.05, were used and variants passing these thresholds were designated as expressed SNVs.

#### Human endogenous retrovirus (HERV) analysis

Expression of previously annotated HERVs (Rooney et al., 2015; Panda et al., 2018; Smith et al., 2018) was analysed. HERV loci used in these three studies were taken from Mayer et al. (2011) and Vargiu et al. (2016) with 66 and 3173 loci respectively. BLASTn was used to match example sequences from HERVs in Mayer et al. to GRCh38, chromosome coordinates with the greatest homology over the greatest length were taken as the best match. The Lift Genome Annotations tool from UCSC (https://genome.ucsc.edu/cgi-bin/hgLiftOver) was used to convert annotated GRCh37 HERV loci coordinates from Vargui et al. to GRCh38 coordinates. Comparing the new coordinates, 47 of the 66 HERVs from Mayer et al. were present in the list of 3173. Coordinates of all the unique elements were then compared to a custom repeat region annotation previously built using the Dfam 2.0 library (v150923) for GRCh38 (Attig et al., 2017). For this custom annotation, different regions of the same provirus (e.g. the LTR and internal genes) were annotated separately, these regions were merged to allow accurate quantitation of reads from the same provirus (Attig et al., 2017). LTR-containing repeat regions from the custom annotation had to begin, end, or be fully contained within previously annotated loci to be considered a match, a buffer of 5 bases either end of the locus was included. Previously annotated HERV loci from Mayer et al. and Vargiu et al. were found to overlap multiple repeat regions per locus in our custom annotations, or were found to overlap no repeat regions at all. Some loci also overlapped other endogenous retroelement types such as LINEs and SINEs, as well as overlapping canonical gene exons. For this analysis, only expression of matching LTR-containing elements was considered rather than expression of all repeats and genes overlapping previously annotated loci. Reads were aligned to GRCh38 using Hisat2 (version 2.1.0), SAMtools (version 1.3.1) was used to convert the output to BAM files. Expression of LTR-containing elements was measured using read counts calculated by the featureCounts function from the Subread package (Liao et al., 2014) (version 1.5.0, with parameters -p -C -B -f -T 2), multi-mapping reads were not counted. Analysis for purified immune cell subset expression were performed on publicly available datasets from Linsley et al. (2014) (E-MTAB-8208 (EMBL-EBI)) and Kazachenka et al. (2019) (accession no. GSE60424 (GEO)). LTR-overlapping transcripts expressed highly specifically in ccRCC were previously described (Attig et al., 2019). These transcripts were identified through *de novo* transcriptome assembly and their expression quantified in by transcript per million calculations, as previously described (Attig et al., 2019).

#### Differential gene expression analysis, pathway analysis and gene set enrichment

DESeq2 (Love et al., 2014) was used for differential expression analysis, using the binomial Wald test after estimation of size factors and estimation of dispersion. To identify genes differentially expressed between responders and non-responders, we considered only transcripts with normalized count number >5 in at least 5 patients. Pathway analysis was performed using the R package XGR (Fang et al., 2016b) using the gene ontology biological process (GOBP) databases. Induced and suppressed transcripts were analysed separately against the background of all tested transcripts. The “lea” ontology algorithm was used.

#### T cell subset gene signature

Gene signature or single gene enrichment was evaluated using RSEM abundance, z score scaled across all samples for which RNA-Seq was available. Signature analysis was performed using 22 immune-related signatures listed below: i) the Danaher immune score is a 60-marker gene signature derived from pan-cancer RNAseq analysis for 14 immune cell populations, where marker genes have been benchmarked against histological tumour-infiltrating lymphocyte (TIL) estimates and flow cytometry data (Rosenthal et al., 2019; Danaher et al., 2017); ii) IMmotion150 (McDermott et al., 2018); iii) Javelin101 (Motzer et al., 2019).

(1) Danaher T cells: CD3D, CD3E, CD3G, CD6, SH2D1A, TRAT1
(2) Danaher CD8: CD8A, CD8B
(3) Danaher Cytotoxic: CTSW, GNLY, GZMA, GZMB, GZMH, KLRB1, KLRD1, KLRK1, PRF1, NKG7
(4) Danaher B cells: BLK, CD19, MS4A1, TNFRSF17, FCRL2, KIAA0125, PNOC, SPIB, TCL1A
(5) Danaher NK cells: NCR1, XCL2, XCL1
(6) Danaher CD45: PTPRC
(7) Danaher DC: CCL13, CD209, HSD11B1
(8) Danaher CD8Ex: CD244, EOMES, LAG3, PTGER4
(9) Danaher Mac: CD163,CD68, CD84, MS4A4A
(10) Danaher Mast: MS4A2,TPSAB1,CPA3,HDC,TPSB2
(11) Danaher Neut: CSF3R, S100A12, CEACAM3, FCAR, FCGR3B, FPR1, SIGLEC5
(12) Danaher NKCD56: IL21R, KIR2DL3, KIR3DL1, KIR3DL2
(13) Danaher Th1: TBX21
(14) Danaher Treg: FOXP3
(15) IMmotion150 Angio: VEGFA, KDR, ESM1, PECAM1, ANGPTL4, CD34
(16) IMmotion150 Teff: CD8A, IFNG, PRF1, EOMES, CD274
(17) IMmotion150 Myeloid: CXCL1, CXCL2, CXCL3, CXCL8, IL6, PTGS2
(18) Javelin101 TCR: CD3G, CD3E, CD8B, THEMIS, TRAT1, GRAP2, CD247
(19) Javelin101 T cell: CD2, CD96, PRF1, CD6, IL7R, ITK, GPR18, EOMES, SIT1, NLRC3
(20) Javelin101 NK: CD2, CD96, PRF1, CD244, KLRD1, SH2D1A
(21) Javelin101 chemo: CCL5, XCL2
(22) Javelin101 other: CST7, GFI1, KCNA3, PSTPIP1
The signature score was calculated as the arithmetic mean of z score scaled expression of all genes in that signature for each sample.

#### TCR sequencing

TCR β-chain sequencing was performed by utilizing whole RNA extracted from tissue samples or from cryopreserved PBMC samples, by using a quantitative experimental and computational TCR sequencing pipeline described previously (Best et al., 2015; Oakes et al., 2017; Thomas et al., 2013; Uddin et al., 2019). An important feature of this protocol is the incorporation of a UMI attached to each cDNA TCR molecule that enables correction for PCR and sequencing errors, which allows higher quantitative precision compared to alternate protocols in the TCR sequences retrieved (Oakes et al., 2017; Barennes et al., 2020). The suite of tools used for TCR identification, error correction and CDR3 extraction is freely available at https://github.com/innate2adaptive/Decombinator.

For each TCR, we computed the abundance as the count of UMIs mapping to this TCR divided by the total number of UMIs in the sample. If several samples were available at a given patient-timepoint pair, the resulting abundance was calculated as the sum of counts for this TCR across the available samples divided by the sum of total counts across these samples.

#### Repertoire similarity measure

The similarity between two TCR repertoires was assessed with the normalised dot product (also known as the cosine similarity) between the vectors of TCR abundance. This measure is a well-established metric widely used in machine learning to compare numerical vectors and gives a value between 0 (no similarity, that is, orthogonal vectors) and 1 (complete similarity, from vectors with an identical magnitude and direction in the feature space). Each pair of repertoires is represented as two vectors of equal length, indexed by the union of TCRs found in both repertoires and containing the number of times each TCR is detected in each of the two repertoires (each position contains an integer ≥0). The similarity between the two vectors is given as

$$Similarity=\frac{TCR1\cdot TCR2}{\left\|TCR1\right\|*\left\|TCR2\right\|}$$

where and are the abundance vectors, represents the vector product and paired vertical bars represent the Euclidean norm of the vector.

For longitudinal similarity (Figures 5D, S7G, and S8B), the similarity measure was performed on the TCR abundance vectors derived from (patient, timepoint) pairs.

For spatial similarity (Figures S7A and S7B), the similarity measure was performed on the TCR abundance vectors derived from each sample within a (patient, timepoint) pair. For this analysis, samples from different timepoints were not compared.

#### Repertoire clonality index

The clonality index was estimated for each sample by using the command entropy from the entropy R package, on the basis of the observed frequency of the TCRs in that sample

$$Clonality\;=1\;-\;\left(\sum p_{i}\times log\;p_{i}\right)/\;ln\;N$$

where $p_{i}$ is the frequency of the ith TCR in the repertoire and is the number of TCRs in that repertoire.

#### Classification of expanded, contracted and persistent TCRs

The difference in abundance between Pre-treatment and On-treatment was calculated with the poisson.test function in R, as the data were counts. TCRs with P values above 0.01 were labelled as persistent.

#### Classification of expanded TCRs

We counted the number of TCRs detected with frequencies above a range of frequency thresholds in the tumour repertoires. To measure how such defined expanded TCRs were representative of the shape of the TCR distribution captured by the clonality score, we computed the prevalence of the expanded population amongst the entire repertoire, for each threshold. To do so, we took the sum of counts for expanded TCRs and divided it by the sum of all counts in the sample. The proportion obtained was then correlated to the matched clonality score with the Spearman’s rank correlation.

To focus on the most expanded TCRs (Figures 5C, 5E, S7E, and S7F), we examined those present above a threshold frequency of 2/1,000 (corresponding to the top 1% of the empirical TCR frequency distribution). At this threshold, which we already described in previously published work (Barennes et al., 2020), the correlation between clonality and proportion of repertoire occupied by expanded TCRs is very strong and the number of TCRs labelled as expanded is greater than for higher thresholds for which this correlation is also significant, which enables to keep the greatest amount of data whilst still applying a stringent filtering step.

#### CDR3 amino acid clustering

The pairwise similarity between pairs of TCRs was measured on the basis of amino acid triplet sharing. Sharing was quantified using the normalized string kernel function stringdot (with parameters stringdot (type = ‘spectrum’, length = 3, normalized = TRUE) from the Kernlab package. The kernel is calculated as the number of amino acid triplets (sets of three consecutive amino acids) shared by two CDR3s, normalized by the number of triplets in each CDR3 being compared. The TCR similarity matrix was converted into a network diagram by using the iGraph package in R. Two TCRs were considered connected if the similarity index was >0.82 (threshold previously optimised in a separate study).

Per (patient, timepoint) pair, we counted the number of clusters containing an expanded CDR3. To normalize the counts of clusters obtained (Nreal) for the input size, for each sample, we randomly selected, outside of the real clustering structure, the number of CDR3s equal to the number of expanded CDR3s in that sample and looked for clusters around those. This control step was repeated 10 times for each (patient, timepoint) pair and we computed the average number of clusters obtained for those control (Ncon) and used Nreal/Ncon as the normalised cluster count value.

We used the clustering structure built as described above for pre-treatment samples and retrospectively labelled expanded clones at that time-point as maintained if they were also expanded post-treatment or as replaced if they were not. By doing so, we could derive the number of pre-treatment clusters containing maintained (resp. replaced) expanded clones which was then divided by the initial count of maintained (resp. replaced) expanded clones present in that sample to obtain the proportion displayed.

#### Frequency ratio

We wanted to capture the rate of clonal replacement that occurs in the tumour repertoires. To do so, for each expanded TCR at baseline that could also be detected after treatment, we computed the ratio of the observed frequency at baseline divided by the observed frequency after-treatment. To derive a metric for each patient, we computed the average of ratio scores obtained for all expanded TCRs at baseline (those that could not be detected after treatment were excluded).

**Analysis of Yost et al. cohort** Bulk TCR sequencing data from Yost et al. (2019) were retrieved from Adaptive Biotechnologies’ ImmuneACCESS database (https://doi.org/10.21417/KY2019NM; https://clients.adaptivebiotech.com/pub/yost-2019-natmed). Intratumoural longitudinal similarity was measured with the cosine metric for 11 patients split between responders and non-responders as defined in their original work. See Table S4 for patient and sample annotations.

#### Multiplex immunofluorescence staining and image analysis

Formalin-fixed paraffin-embedded (FFPE) blocks were cut in 2 micron thick slides. The slides were baked for 60 minutes and stained using the antibodies listed below and opal fluorophores. Leica Bond III machine was used for the immunofluorescence staining. Images of the stained slides were acquired by using the Vectra 3 automated quantitative pathology imaging system (Akoya Biosciences). Matching haematoxylin and eosin (H&E) image of each slide was reviewed by a pathologist and areas to annotate on the immunofluorescent images for analysis were identified. Necrotic and stromal areas as well as non-tumour areas were excluded and tumour areas were scored. Slides for patient ADR009 were not evaluable due to necrosis. Total of 61 samples (41 pre-treatment and 20 on treatment samples) for the first mIF panel and 60 samples (40 pre-treatment and 20 on treatment samples) were for the second IF panel were used for analysis. The following antibodies were used for mIF staining: CD3 (Mouse monoclonal, LN10, 1:100 dilution on Opal 520 in 1:50 dilution), CD4 (Mouse monoclonal, 4B12, 1:50 dilution on Opal 540 in 1:100 dilution), CD8 (Mouse monoclonal, 4B11, 1:100 dilution on Opal 540 in 1:150 dilution and on Opal 620 in 1:150 dilution), FOXP3 (Mouse Monoclonal, 236A/E7, 1:80 dilution on Opal 570 in 1:150 dilution), CD163 (Mouse monoclonal, 10D6, 1:100 dilution on Opal 690 in 1:50 dilution), Granzyme B (Mouse monoclonal, 11F1, 1:80 dilution, on Opal 620 in 1:150 dilution)

Up to 25 multispectral images (MSI) were acquired per slide depending on the size of the tumour to include all representative areas of the tumour. Representative MSIs from different slides were used while training the algorithms for each marker. Scoring of each slide was performed using the inForm software on Vectra. The quality and accuracy of the scoring was checked by two clinicians one of whom was a histopathologist. MSIs with poor tissue quality were excluded from the analysis. Merged data obtained by using the inForm software was analysed using the phenoptrReports tool (Akoya Biosciences) on R. T cells subsets (CD8^+^, CD4^+^ effectors, Tregs and CD8^+^CD4^+^ double positive cells) were scored both out of total cells counted on each slide and out of the total T cells counted. CD163 cells were scored out of total cells counted per slide. Overall granzyme expression was scored in relation to the total T cell and CD163^+^ cell count. Granzyme B expression on CD8^+^ cells was scored out of the total CD8^+^ cells. Median scoring value was used for each patient per time point and two-sided Mann-Whitney U test was used for statistical analysis of the data.

The mIF and mIHC antibody panels were designed to evaluate T cell subsets, B cells, myeloid cells, and GZMB expression. This was conducted given 1) double positive (CD8^+^CD4^+^) T cells with high degrees of TCR clonality have previously been described in ccRCC (Menard et al., 2018); 2) myeloid inflammation has been associated with blunting of anti-tumour T cell activity in metastatic ccRCC (McDermott et al., 2018); and 3) high tumor infiltration with B cells and plasma cells have previously been shown to correlate with favourable clinical outcomes across cancer types (Berntsson et al., 2016; Kroeger et al., 2016; Yeong et al., 2018).

#### Immunohistochemistry

FFPE tissue sections of clear cell renal cell carcinoma and normal tonsil tissues were subjected to H&E and multiplex immunostaining. The primary antibodies used for multiplex immunolabeling are as follows: CD19 (rabbit monoclonal, SP291, 1:10 dilution), CD138 (mouse monoclonal, MI15, 1:100 dilution), PD-1 (mouse monoclonal, NAT105/E3, 1:2 dilution). *MLH1* (mouse monoclonal, WH0004292M2, 1:750 dilution) and *B2M* (rabbit monoclonal, 4H5L6, 1:500 dilution) were used as single stains on tissue from ADR015 separately.

To establish optimal staining conditions each antibody was tested and optimized on 2–4 um cut tissue sections of human reactive tonsil and normal kidney by applying conventional single immunohistochemistry. In brief sections were de-waxed and re-hydrated prior to the multiplex immunolabeling whose procedure was adapted and performed according to the established protocol described elsewhere (Marafioti et al., 2003). Total of 59 samples (40 pre-treatment and 19 on treatment samples) for the mIHC panel.

#### Staining assessment and data handling

Specificity of the staining was assessed by a haematopathologist with expertise in multiplex-immunostaining. Scanned slide images were obtained with the use of NanoZoomer Digital Pathology System (Hamamatsu, Japan). Total of 60 samples (41 pre-treatment and 19 on treatment samples) were used for analysis.

#### Flow cytometry

Renal tumour resections and normal tissue were cut into small pieces (2-3mm) by using sterile disposable scalpel plus forceps in RPMI (Sigma-Aldrich) with Collagenase I (Sigma-Aldrich) (for ADR013 tumour and normal tissue), Liberase (for ADR001 tumour tissue) and DNAse I (Roche) and was digested for 1 hour at room temperature using the gentleMACS dissociator (Miltenyi Biotec). The digest was passed through a 70-μm cell strainer by using 5-10 ml of RPMI containing 2% fetal bovine serum (FBS) to obtain a single-cell suspension. Lymphocytes were obtained from the single-cell suspension by using Ficoll Paque Plus (GE Healthcare) density gradient centrifugation (750g for 10 min). Isolated lymphocytes were washed with RPMI and 2%FBS and cryopreserved in 90% FBS with 10% dimethyl sulfoxide (Sigma–Aldrich). PBMCs were isolated from blood samples collected in Vacutainer EDTA blood collection tubes (BD) using Ficoll Paque Plus (GE Healthcare) density gradient centrifugation and cryopreserved in in 90% FBS with 10% dimethyl sulfoxide (Sigma–Aldrich).

Thawed lymphocytes were washed with 1× phosphate-buffered saline (PBS) and were stained with the antibodies listed below. Antibody mastermixes were prepared in Brilliant Staining Buffer (BD). eBioscience™ Foxp3/Transcription Factor Staining Buffer Set was used for the intracellular staining. Samples were stained using the following antibodies: CD8 (RPA-T8, BUV496), CD45RA (HI100, BUV563), CD4 (SK3, BUV615), CD38 (HIT2, BUV737), CD3 (SK7, BV705), FOXP3 (206D, BV421), CCR4 (L291H4, BV510), Viability dye (Yellow Fluorescent reactive dye, BV570), CD57 (QA17A04, BV605), Ki67 (B56, BV650), CD39 (TU66, BV711), CCR7 (G043H7, BV750), CD69(FN50, BV785), CD103 (Ber-ACT8, BB515), CXCR5 (RF8B2, PerCp-Cy5-5), TCF-7 (7F11A10, PE), Granzyme B (GB11, PE-CF594), CD25 (M-A251, PE-Cy5), PD-1 (EH12.2H7, PE-Cy7), TOX (REA473, APC), IgG4 (Biotin), 4-1BB (4B4-1, BUV661), TIM-3 (7D3, BV650), Streptavidin (BUV395). The samples were acquired on the BD Symphony flow cytometer. Data was analysed using the FlowJo (version 10).

#### PD-1 competition binding assay to evaluate anti-PD1 monoclonal antibody binding

PBMC isolated from healthy individuals were activated *in vitro* using plate coated anti-CD3 and soluble anti-CD28 with 100IU IL-2 per well. 50ul (5ug/mL solution) anti-CD3 was used to coat wells of a 96 well plate which was kept at 4°C overnight. Two washes using 200ul of PBS were performed to remove unbound antibodies the next day. Subsequently, 2 × 10^5^ PBMC were added into each well with subsequent addition of soluble anti-CD28 (2ug/mL). The plate was placed into a humidified 37°C incubator for 72 hours. Following this period, the wells containing activated PBMC were either incubated with 50ul (2.5mg) pembrolizumab or PBS control for 30 minutes. PBS washes were used to remove unbound therapeutic antibodies. Flow cytometric staining of CD3, PD1 and anti-IgG4 was performed thereafter.

#### Single-cell RNA/TCR sequencing

Tumour infiltrating lymphocytes from ADR001 and AD013 were stained with CD3 (PE, SK7 clone), IgG4 (Biotinylated) and Streptavidin (BV650) antibodies for flow cytometry. Stained cells were FACS sorted as CD3^+^IgG4^-^ (40,000 cells) and CD3^+^IgG4^+^ (20,000 cells) for ADR001 and CD3^+^IgG4^-^ (50,000 cells) and CD3^+^IgG4^+^ (90,000 cells) for ADR013. FACS sorted cells were single-cell sorted using the 10X Genomic machine. The sorted cells were processed using the 10X Genomic Chromium Next GEM Single Cell 5’ Reagents Kit V2 (dual index) for 5’gene expression library construction and V(D)J library construction. The samples were sequenced on the NextSeq using the High Output Kit v2.5 (150 Cycles).

FASTQ files containing gene expression (GEX) and VDJ were demultiplexed using cellranger mkfastq (10x Genomics). GEX reads were aligned to GRCh38 and counted using cellranger count, VDJ reads were aligned to cellranger’s GRCh38 VDJ reference dataset using cellranger vdj. Expression matrices were analysed using the Seurat package (Stuart et al., 2019). To remove technical variation in the data, TCR, ribosomal and heat-shock protein genes were removed from the analysis, also cells with mitochondrial reads making up >10% total read content were removed. 8382 CD3^+^IgG4^-^ and 10083 CD3^+^IgG4^+^ cells in ADR013; and 4648 CD3^+^IgG4^-^ and 3343 CD3^+^IgG4^+^ cells in ADR001 were retained after quality control filtering. Datasets were integrated using SCTransform integration (Hafemeister and Satija, 2019) using the recommended parameters and regressing the % mitochondrial read content. Principal component analysis (PCA) and uniform manifold approximation and projection (UMAP) dimensional reduction (dims = 1:30) was then performed using RunPCA and RunUMAP. Publicly available gene signatures for T cell states were obtained from the following publications: Schietinger et al. (2016), Thommen et al. (2018), Guo et al. (2018), Li et al. (2019a), Yost et al. (2019), Miller et al. (2019), Zhou et al. (2010), and Litchfield et al. (2021) (Table S4). The proportion of reads mapping to the genes in each signature for each cell was then calculated using PercentageFeatureSet. All differential gene expression analysis were carried out on log normalised gene expression values (using NormalizeData, default parameters) using the MAST algorithm (Finak et al., 2015) within FindMarkers. GOBP analysis was carried out using the XGR package (Fang et al., 2016b) using the “lea” algorithm. scTCR data was analysed using scRepertoire. Cells were considered of the same clone if they contained a matching TRB sequence and CDR3 gene.

#### scRNA/TCRseq meta-analysis

Raw count matrices and scTCR annotations were downloaded from Braun et al. (2021) (downloaded from the published supplemental data), Borcherding et al. (2021) (Gene Expression Omnibus accession: GSE121638) and Krishna et al. (2021) (https://trace.ncbi.nlm.nih.gov/Traces/sra/sra.cgi?analysis=SRZ190804). Cells and genes in the ADR001 and ADR013 samples were filtered as described previously. All samples were then filtered for CD8, CD4 and Tregs using expression cutoffs (counts ≥1 considered positive for each gene; CD8 = CD8A^+^CD4^−^FOXP3^-^, CD4 = CD8A^−^CD4^+^FOXP3^-^, Treg = CD8A^−^CD4^+^FOXP3^+^). All samples were merged into a single Seurat object, which was then processed using NormalizeData and FindVariableFeatures (default settings), then ScaleData with % mitochondrial transcript being regressed, followed by RunPCA. Harmony based integration (Korsunsky et al., 2019) was then used (through the Seurat wrapper RunHarmony) to batch correct the samples (patient was used as the batch variable, kmeans_init_nstart = 20, kmeans_init_iter_max = 100). Harmony integration was chosen over Seurat integration (used in the ADR001 and ADR013 analysis) due to Harmony’s better performance with numerous batches. RunUMAP was then run using the Harmony reduction and dims = 1:30. All differential gene expression analysis were carried out using the MAST algorithm (Finak et al., 2015) within FindMarkers. TCR expansion was calculated. TCR expansion was calculated (based on TRB only) for each patient using the “expa” metric from STARTRAC (Zhang et al., 2018b).

### Quantification and statistical analysis

Statistical analysis was performed in R and GraphPad Prism 8. Correlation was carried out with the Spearman’s rank correlation test. We used mixed effect modelling when appropriate. We used the Mann–Whitney two-tailed paired or non-paired nonparametric tests (as appropriate) to determine whether two independent samples were selected from the same population. P values were considered significant if less than 0.05, and significance values were corrected for multiple testing by Bonferroni correction when appropriate. High dimensional flow cytometry analysis was performed using FlowJo 10. Analyses and visualization of HERV expression were additionally performed in Qlucore Omics Explorer (Qlucore, Lund, Sweden). Data visualization was performed in BioRender, R and GraphPad Prism 8.

### Additional resources

Clinical trial registry numbers:

ADAPTeR: https://clinicaltrials.gov/ct2/show/NCT02446860.

TRACERx Renal: https://clinicaltrials.gov/ct2/show/NCT03226886.

PEACE: https://clinicaltrials.gov/ct2/show/NCT03004755.

## Consortia

### The TRACERx Renal Consortium

Lewis Au, Ben Challacombe, Ashish Chandra, Simon Chowdhury, William Drake, Archana Fernando, Nicos Fotiadis, Andrew Furness, Emine Hatipoglu, Karen Harrison-Phipps, Steve Hazell, Peter Hill, Catherine Horsfield, James Larkin, Jose I. Lopez, Teresa Marafioti, David Nicol, Tim O’Brien, Jonathon Olsburgh, Lisa Pickering, Alexander Polson, Sergio Quezada, Sarah Rudman, Scott Shepherd, Charles Swanton, Samra Turajlic, Mary Varia, Hema Verma.

### The PEACE Consortium

Chris Abbosh, Kai-Keen Shiu, John Bridgewater, Daniel Hochhauser, Martin Forster, Siow-Ming Lee, Tanya Ahmad, Dionysis Papadatos-Pastos, Sam Janes, Peter Van Loo, Katey Enfield, Nicholas McGranahan, Ariana Huebner, Sergio Quezada, Stephan Beck, Peter Parker, Henning Walczak, Tariq Enver, Rob Hynds, Mary Falzon, Ian Proctor, Ron Sinclair, Chi-wah Lok, Zoe Rhodes, David Moore, Teresa Marafioti, Elaine Borg, Miriam Mitchison, Reena Khiroya, Giorgia Trevisan, Peter Ellery, Mark Linch, Sebastian Brandner, Crispin Hiley, Selvaraju Veeriah, Maryam Razaq, Heather Shaw, Gert Attard, Mita Afroza Akther, Cristina Naceur-Lombardelli, Lizi Manzano, Maise Al-Bakir, Simranpreet Summan, Nnenna Kanu, Sophie Ward, Uzma Asghar, Emilia Lim, Faye Gishen, Adrian Tookman, Paddy Stone, Caroline Stirling, Lewis Au, Andrew Furness, Kim Edmonds, Nikki Hunter, Sarah Sarker, Sarah Vaughan, Mary Mangwende, Karla Lingard, Lavinia Spain, Scott Shepherd, Haixi Yan, Ben Shum, Eleanor Carlyle, Steve Hazell, Annika Fendler, Fiona Byrne, Nadia Yousaf, Sanjay Popat, Olivia Curtis, Gordon Stamp, Antonia Toncheva, Emma Nye, Aida Murra, Justine Korteweg, Nahid Sheikh, Debra Josephs, Ashish Chandra, James Spicer, Ula Mahadeva, Anna Green, Ruby Stewart, Lara-Rose Iredale, Tina Mackay, Ben Deakin, Debra Enting, Sarah Rudman, Sharmistha Ghosh, Lena Karapagniotou, Elias Pintus, Andrew Tutt, Sarah Howlett, Vasiliki Michalarea, James Brenton, Carlos Caldas, Rebecca Fitzgerald, Merche Jimenez-Linan, Elena Provenzano, Alison Cluroe, Grant Stewart, Colin Watts, Richard Gilbertson, Ultan McDermott, Simon Tavare, Emma Beddowes, Patricia Roxburgh, Andrew Biankin, Anthony Chalmers, Sioban Fraser, Karin Oien, Andrew Kidd, Kevin Blyth, Matt Krebs, Fiona Blackhall, Yvonne Summers, Caroline Dive, Richard Marais, Fabio Gomes, Mat Carter, Jo Dransfield, John Le Quesne, Dean Fennell, Jacqui Shaw, Babu Naidu, Shobhit Baijal, Bruce Tanchel, Gerald Langman, Andrew Robinson, Martin Collard, Peter Cockcroft, Charlotte Ferris, Hollie Bancroft, Amy Kerr, Gary Middleton, Joanne Webb, Salma Kadiri, Peter Colloby, Bernard Olisemeke, Rodelaine Wilson, Ian Tomlinson, Sanjay Jogai, Christian Ottensmeier, David Harrison, Massimo Loda, Adrienne Flanagan, Mairead McKenzie, Allan Hackshaw, Jonathan Ledermann, Kitty Chan, Abby Sharp, Laura Farrelly, and Hayley Bridger.

## Data Availability

Raw bulk whole-exome sequencing, RNA-seq, and TCR-seq data have been deposited to the European Genome-phenome Archive (Accession numbers EGAS00001005638, EGAD00001008163, EGAD00001008165, respectively). Raw data for the single-cell RNA and TCR-seq experiments have been deposited (EGAD00001008166). To facilitate ease of use, we have also deposited single-cell count matrices, VDJ annotations, and metadata (https://doi.org/10.5522/04/16573640.v1), and processed bulk-TCR sequence data (https://doi.org/10.5522/04/16571573.v1). Clinical data were obtained from the following sources: Yost et al. cohort (Yost et al., 2019); Braun et al. cohort (Braun et al., 2021); Borcherding et al. cohort (Borcherding et al., 2021); Krishna et al. cohort (Krishna et al., 2021).

## Supplemental information

Document S1. Figures S1–S14 and Table S1

Table S2. Sample characteristics for: whole-exome sequencing (including sequencing metrics, TMB, INDEL and neoantigen burden, purity and cancer cell fraction); RNA sequencing; TCR sequencing; and mIF/IHC, related to Figure 1

Table S3. Previously annotated HERV loci matched to custom repeat region annotations, related to Figure 2

Table S4. Clinical metadata for the Yost et al. patient cohort, related to Figure 5

Table S5. List of genes in T cell-specific expression signatures, related to Figure 6

Table S6. Clinical response, sample characteristics, and CD8/CD4/Treg/TCR counts for the meta-analysis of the Braun et al., Krishna et al., and Borcherding et al. datasets combined with data from ADAPTeR, related to Figures 7 and S12

Document S2. Article plus supplemental information
