# Supplementary material for: Determinants of anti-PD-1 response and resistance in clear cell renal cell carcinoma
Source: Cancer Cell. 2021 Nov 8;39(11):1497–1518.e11. doi: 10.1016/j.ccell.2021.10.001 (PMC8599450; doi:10.1016/j.ccell.2021.10.001)
Supplement: Document S1. Figures S1–S14 and Table S1 [file mmc1.pdf]

Cancer Cell, Volume *39*

Supplemental information

Determinants of anti-PD-1 response and resistance in clear cell renal cell carcinoma

Lewis Au, Emine Hatipoglu, Marc Robert de Massy, Kevin Litchfield, Gordon Beattie, Andrew Rowan, Desiree Schnidrig, Rachael Thompson, Fiona Byrne, Stuart Horswell, Nicos Fotiadis, Steve Hazell, David Nicol, Scott T.C. Shepherd, Annika Fendler, Robert Mason, Lyra Del Rosario, Kim Edmonds, Karla Lingard, Sarah Sarker, Mary Mangwende, Eleanor Carlyle, Jan Attig, Kroopa Joshi, Imran Uddin, Pablo D. Becker, Mariana Werner Sunderland, Ayse Akarca, Ignazio Puccio, William W. Yang, Tom Lund, Kim Dhillon, Marcos Duran Vasquez, Ehsan Ghorani, Hang Xu, Charlotte Spencer, José I. López, Anna Green, Ula Mahadeva, Elaine Borg, Miriam Mitchison, David A. Moore, Ian Proctor, Mary Falzon, Lisa Pickering, Andrew J.S. Furness, James L. Reading, Roberto Salgado, Teresa Marafioti, Mariam Jamal-Hanjani, on behalf of the PEACE Consortium, George Kassiotis, Benny Chain, James Larkin, Charles Swanton, Sergio A. Quezada, Samra Turajlic, and on behalf of the TRACERx Renal Consortium

Supplemental Material

Supplementary Table

Table S1. Baseline demographics and patient characteristics and correlations with nivolumab response, and sample annotations, related to Figure 1.

	All patients n=15	Responders n=5	Non-Responders n=10	p-value*
Demographics				
Age, median (range), years	56	56	54	0.84
Male, n (%)	13 (87)	9 (90)	4 (80)	0.59
ECOG, n (%)				
0	8 (53)	3 (30)	5 (50)	0.71
1	7 (70)	2 (40)	5 (50)	
Predominant clear-cell histology, n (%)				
Sarcomatoid/rhabdoid component, n (%)	2 (13)	2 (40)	0 (0)	0.28
Prior nephrectomy, n (%)	6 (40)	3 (30)	3 (60)	0.59
On-treatment nephrectomy, n (%)	2 (13)	1 (10)	1 (20)	0.59
IMDC risk categories, n (%)				
Favourable (0)	2 (13)	1 (20)	1 (10)	
Intermediate (1)	3 (20)	1 (20)	2 (20)	0.61
Poor (≥2)	10 (66)	3 (60)	7 (70)	
Outcomes				
Dead, n (%)	6 (40)	1 (20)	5 (50)	0.26
Best Response by RECIST v1.1				
Complete response	0 (0)	0 (0)	0 (0)	
Partial response	5 (33)	4 (80)	1 (10)	NA
Stable disease	6 (40)	1 (20)	5 (50)	
Progressive disease	4 (27)	NA	1 (10)	
PFS, months, median (range)	4.1	NR (8.4 to NR)	3.3 (1.4 to 5.9)	0.0006
OS, months, median (range)	22.2	NR (12.4 to NR)	22.2 (7.2 to NR)	0.59

*Significance tests: Chi-squared test of categorical variables and Mann-Whitney U test for comparison of median values (responders vs. non-responders).
Percentages may not total 100 because of rounding.
NA - not applicable; ECOG - Eastern Cooperative Oncology Group performance scale; IMDC - International Metastatic Renal-Cell Carcinoma Database Consortium; RECIST - Response Evaluation Criteria In Solid Tumors; PFS - progression free survival; OS - overall survival

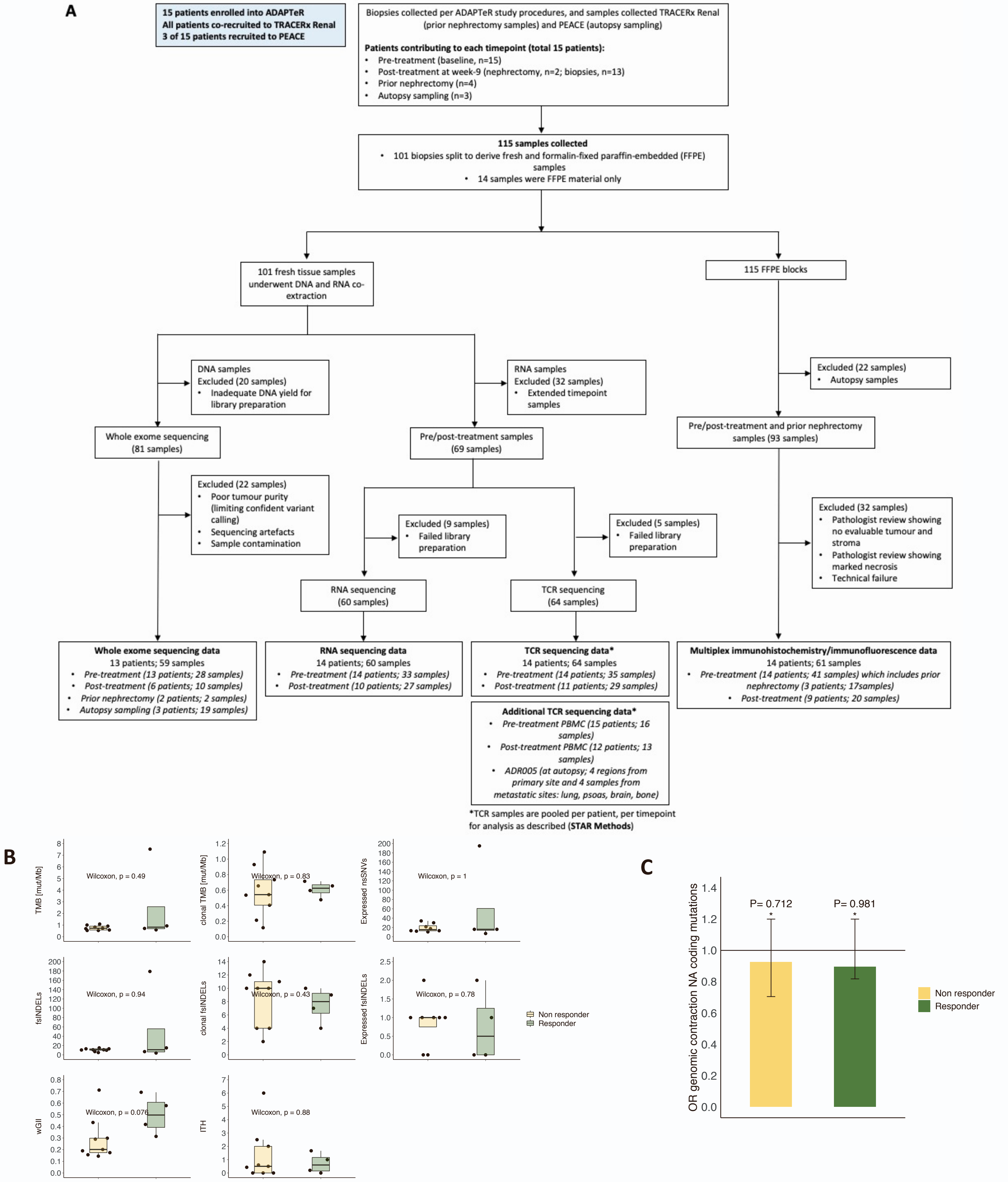

Figure S1. Samples overview, and correlations between nivolumab response and mutational features, related to Figures 1-5.

(A) Consort diagrams for samples that underwent whole-exome sequencing, RNAseq, TCRseq, and multiplex immunofluorescence or immunohistochemistry analyses. **(B)** Boxplots showing no significant correlation between (clonal) TMB, (clonal) fsINDEL load, expressed nsSNVs/fsINDELs, wGII, and ITH to nivolumab response. **(C)** Odds-ratio of neoantigen-encoding mutations to undergo mutation depletion (‘genomic contraction’) compared to the remaining non-synonymous mutations. Mann–Whitney test was performed. P-value >0.05 considered not significant.

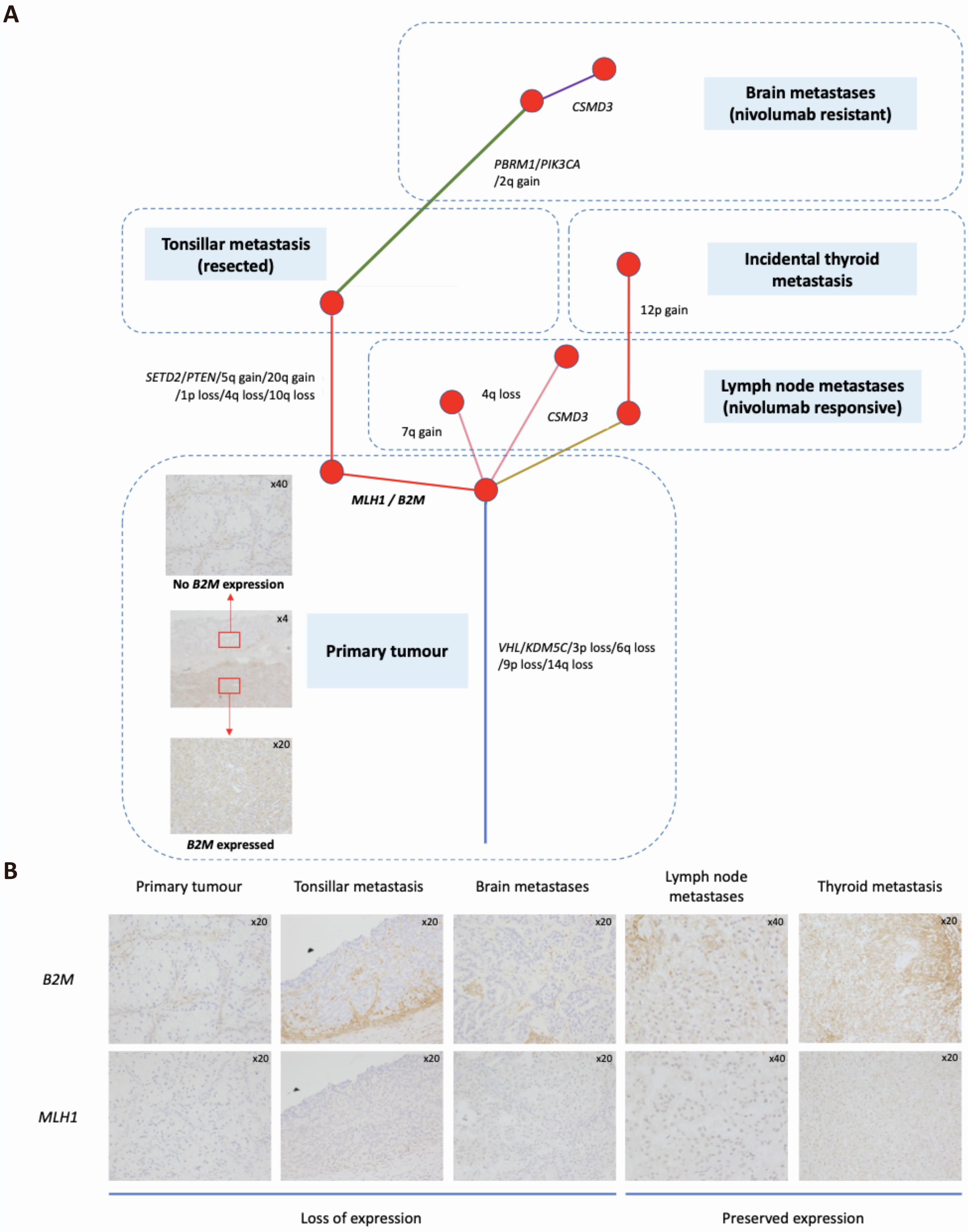

Figure S2. Phylogenetic tree with clinical annotations of ADR015 showing pre-/post-treatment, post-mortem samples, and evolution of metastatic disease, related to Figure 1.

(A) Phylogenetic relationships of the tumor regions. Primary tumour resected five years prior to ADAPTeR study entry, relapsed tonsillar metastasis which was resected and sampled, and nivolumab-responsive and –resistant sites sampled at post-mortem are as annotated. Branch lengths are arbitrary. Driver mutations and somatic copy number alteration events were acquired as annotated by the branch. Primary tumour shows with a small area (subclonal) loss of *B2M* expression as demonstrated by immunohistochemistry staining. **(B)** Immunohistochemistry stains for *MLH1* and *B2M* expression in tumour samples collected at post-mortem.

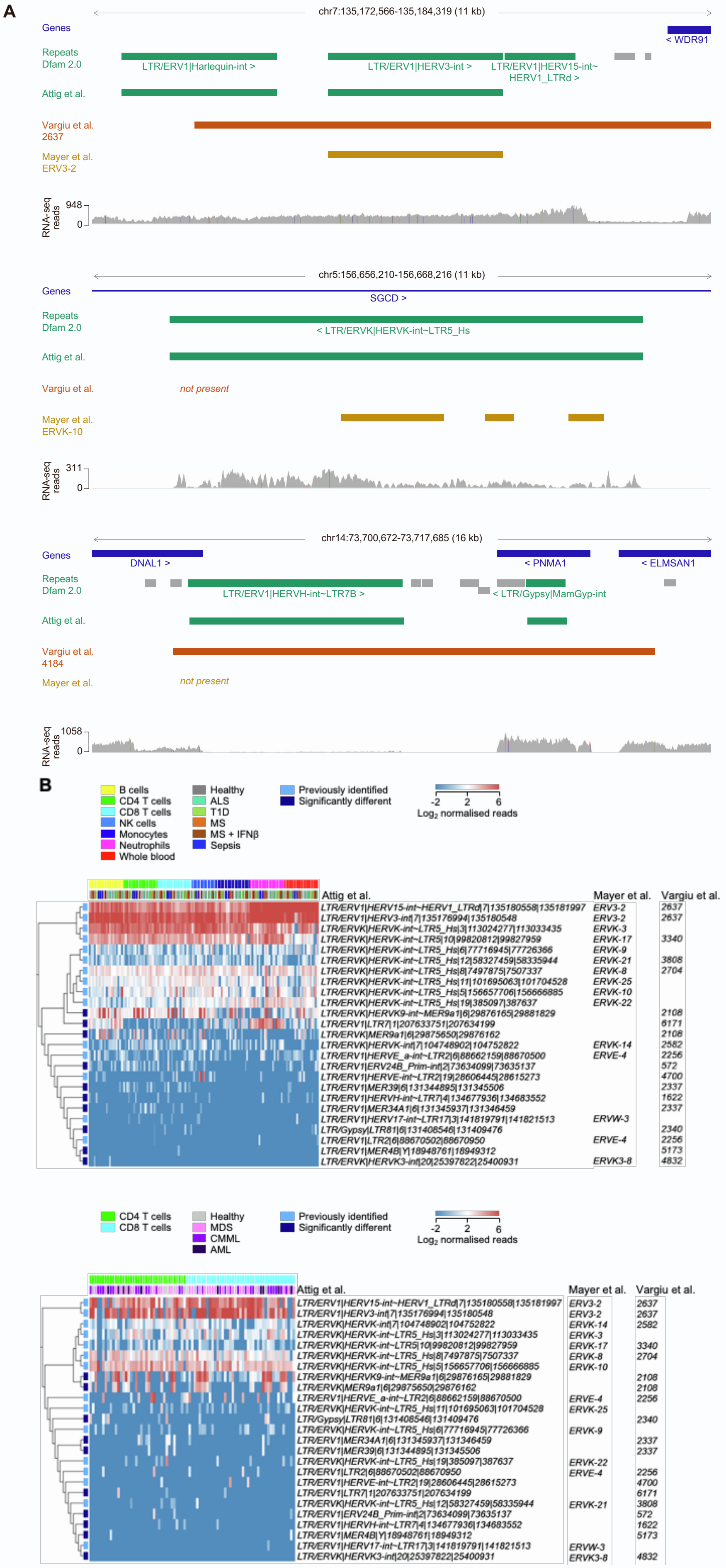

Figure S3. Correspondence of HERV annotation and expression in immune cell types, related to Figure 2

(A) Comparison of HERV annotations by Attig *et al*., Vargiu *et al*. and Mayer *et al*. Three examples are shown, depicting the position of GENCODE annotated genes, Dfam 2.0 annotated repeats and a representative RNAseq read pileup. **(B)** Expression of HERVs previously associated with ccRCC or with nivolumab response, in the indicated purified immune cell types from public RNAseq datasets GSE60424 (top) and E-MTAB-8208 (bottom). Note the expression of LTR/ERVK|HERVK9-int~MER9a1|6|29876165|29881829 integration within the HLA locus in most immune cell subsets and of the LTR/ERV1|LTR7|1|207633751|207634199 integration in neutrophils.

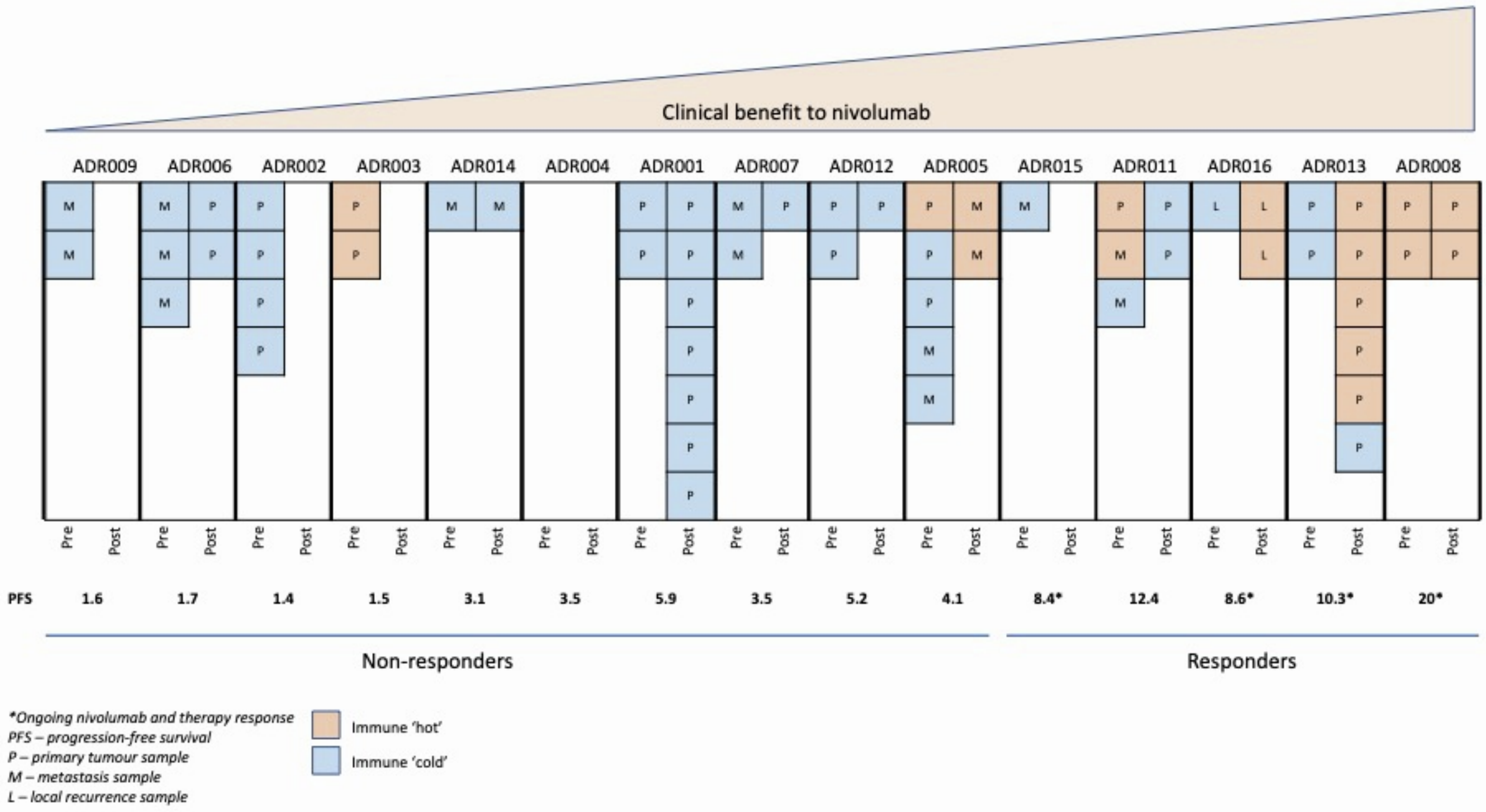

Figure S4. Schematic diagram demonstrating biopsy samples taken pre- and post-treatment classified as immune 'hot/cold' by RNAseq, related to Figure 3

See **Figure 3** for details of RNAseq analysis. Clinical responses are as indicated by progression-free survival. Four responders continue to receive nivolumab at data-lock. PFS – progression-free survival; Pre – pre-treatment tumour sample(s); Post – post-treatment tumour sample(s).

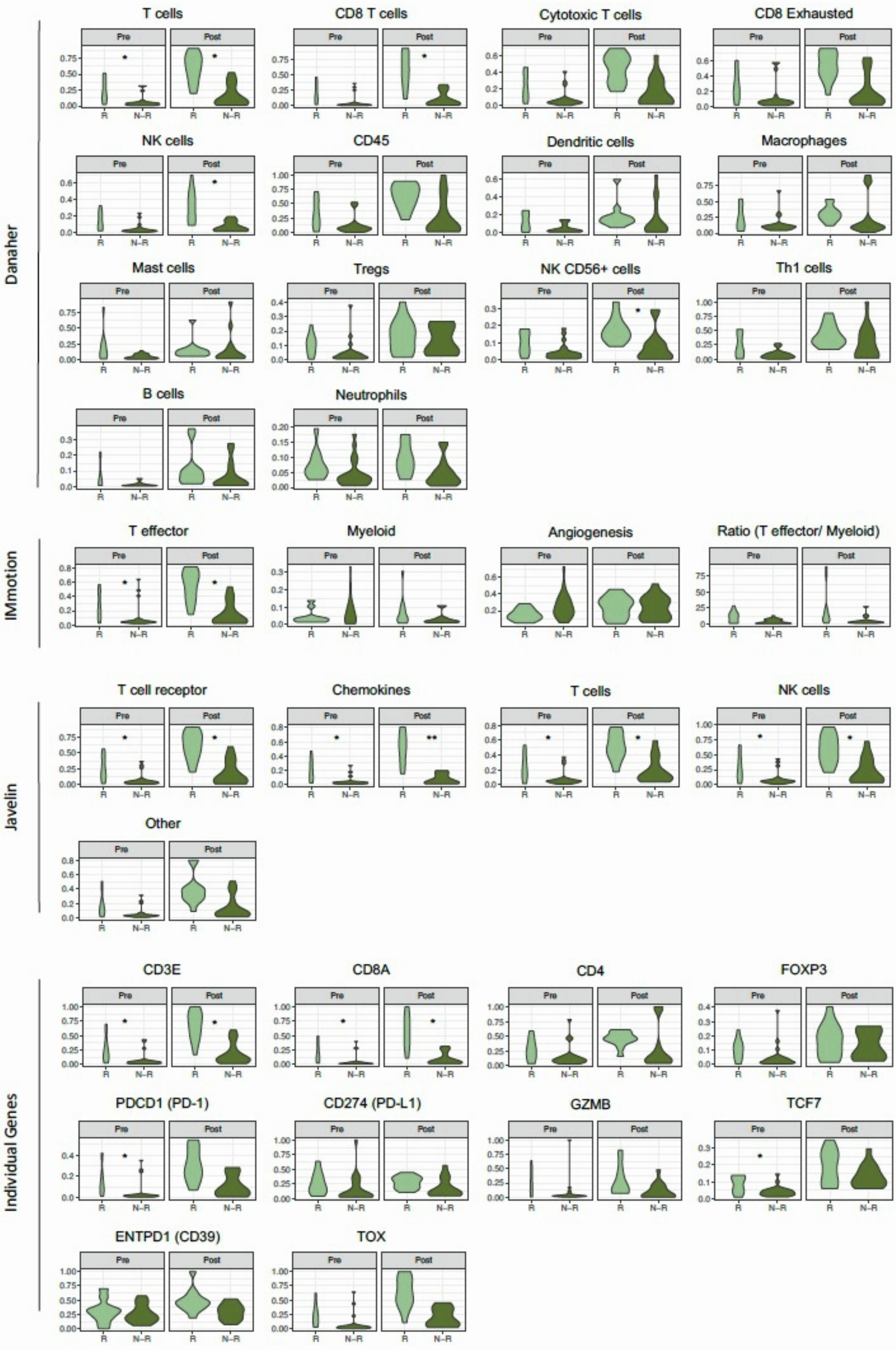

Figure S5. Violin plots comparing response groups at both timepoints by Danaher, IMMOTION150, Javelin101 signatures and by individual gene expression, related to Figure 3

See **STAR Methods** for details of signature analysis. The two-sided Mann–Whitney test performed on one value per patient (score averaged by median value across biopsies if several available at a given time point), significant *P* value are indicated (*: *P*<0.05; **: *P*<0.01). *R - responders; N-R - non-responders.*

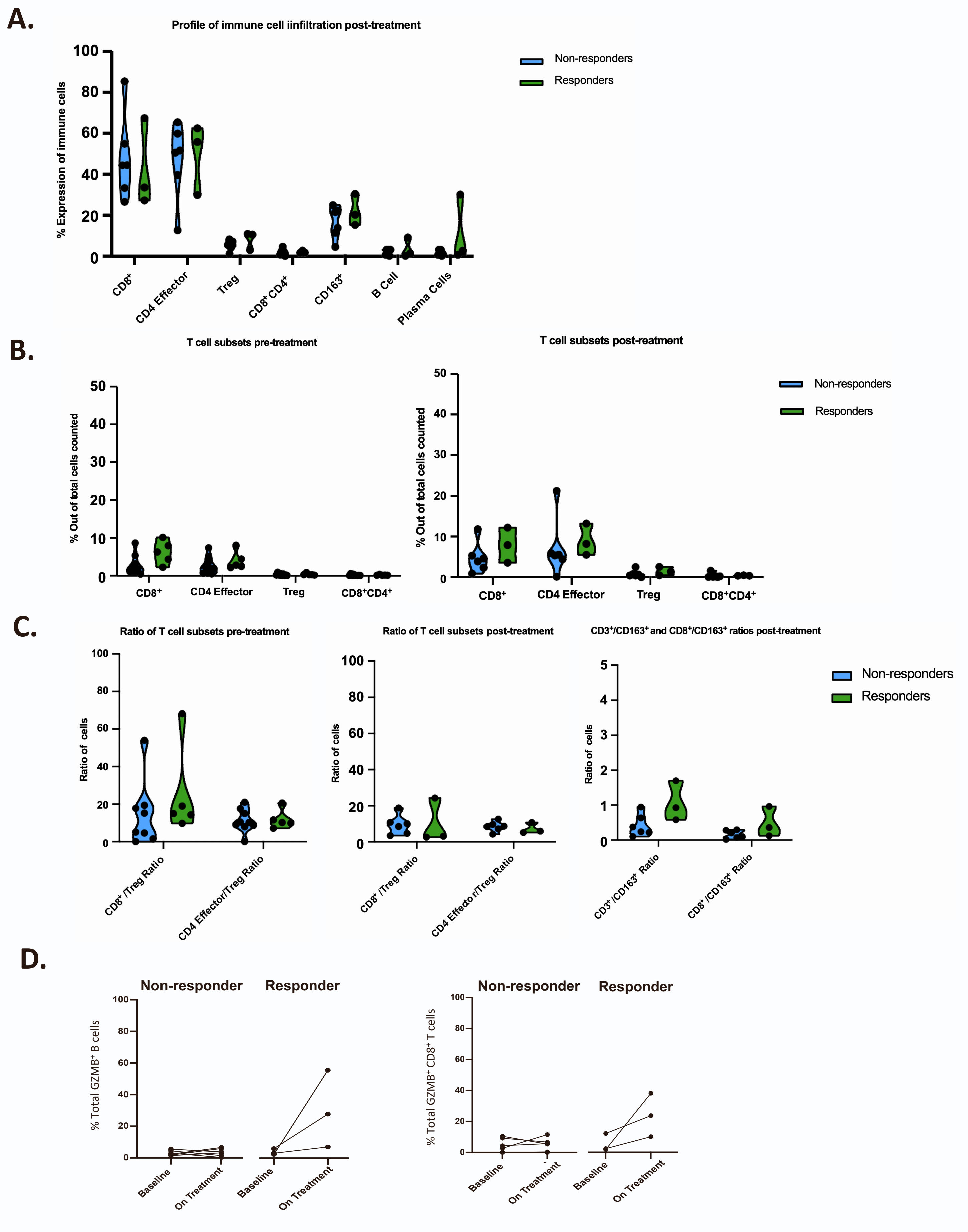

Figure S6. Immune cells subset comparisons of pre- and post-treatment samples, related to Figure 4

(A) Immune cell subset expression levels in non-responders and responders on treatment are shown. **(B)** Expression level of T cell subsets out of total cells counted is shown. **(C)** Ratio of T cells subsets in non-responders and responders at baseline and on treatment; $CD3^+$ T cells to $CD163^+$ myeloid cells, and $CD8^+$ T cells to $CD163^+$ myeloid cell ratios between responders and non-responders on treatment is shown. **(D)** Change in total GZMB expression and on $CD8^+$ T cells from consecutive biopsies from six non-responder patients and three responder patients.

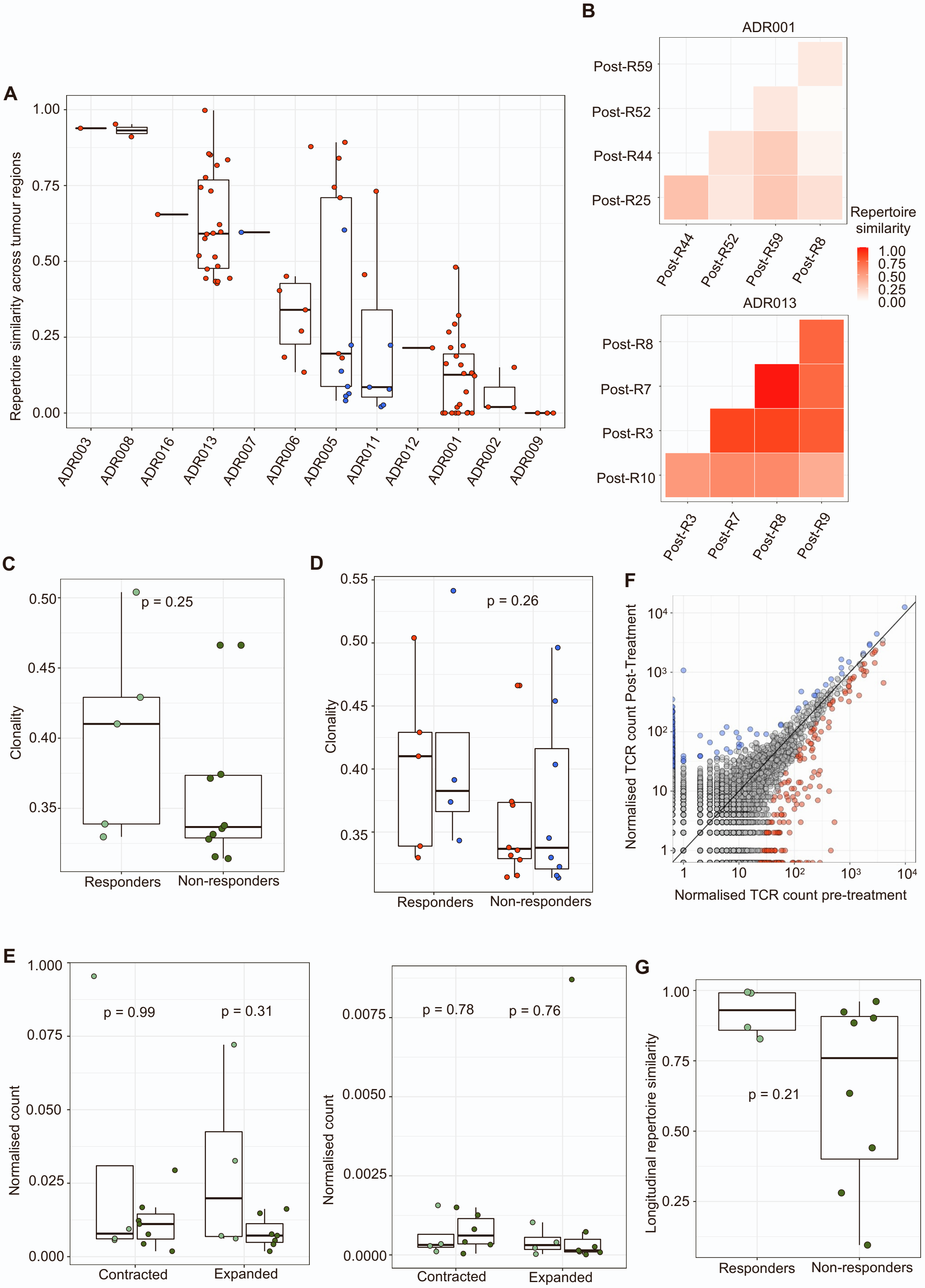

Figure S7. Clonotype dynamics in PBMC and intra- and inter-patient TCR repertoire heterogeneity, related to Figure 5

(A) The TCR repertoires of multiple biopsies from a patient's tumour were sequenced and a pairwise comparison of the repertoires of different biopsies from the same timepoint was performed by using the cosine metric (**STAR Methods**). The pairwise intratumoral TCR repertoire similarity is shown for each patient. Each circle represents a comparison between two samples from the same patient (n = 87 total comparisons from 12 patients). Red (resp. blue) circles indicate a pair of biopsies originating from the same site (resp. different metastatic sites). **(B)** Heat maps showing the pairwise similarities of a selection of 5 biopsies in the post-treatment nephrectomy for ADR001 (top) and ADR013 (bottom). Biopsies were selected based on comparable TCR counts. **(C)** The intratumoural clonality scores post-treatment are shown for each patient. **(D)** The peripheral TCR repertoire clonality score pre-treatment and on-treatment is shown for each patient. Patients are split between responders and non-responders. Mixed-effect model *P* value shown. **(E)** The number of intratumoral (left panel) and peripheral (right panel) clones labelled as expanded or contracted between timepoints, per patient, normalized for the total number of clones tested. Two-sided Mann–Whitney tests *P* value shown. Light green (responders); dark green (non-responders). **(F)** Correlated clone sizes in blood samples. Scatter plots of blood clone size after treatment and before treatment are shown for all patients. Clones are coloured by expansion/contraction status (**STAR Methods**). **(G)** The peripheral cosine score between pre-treatment and on-treatment is shown for each patient. Patients are split between responders and non-responders. Two-sided Mann–Whitney test *P* value shown; n=12 patients.

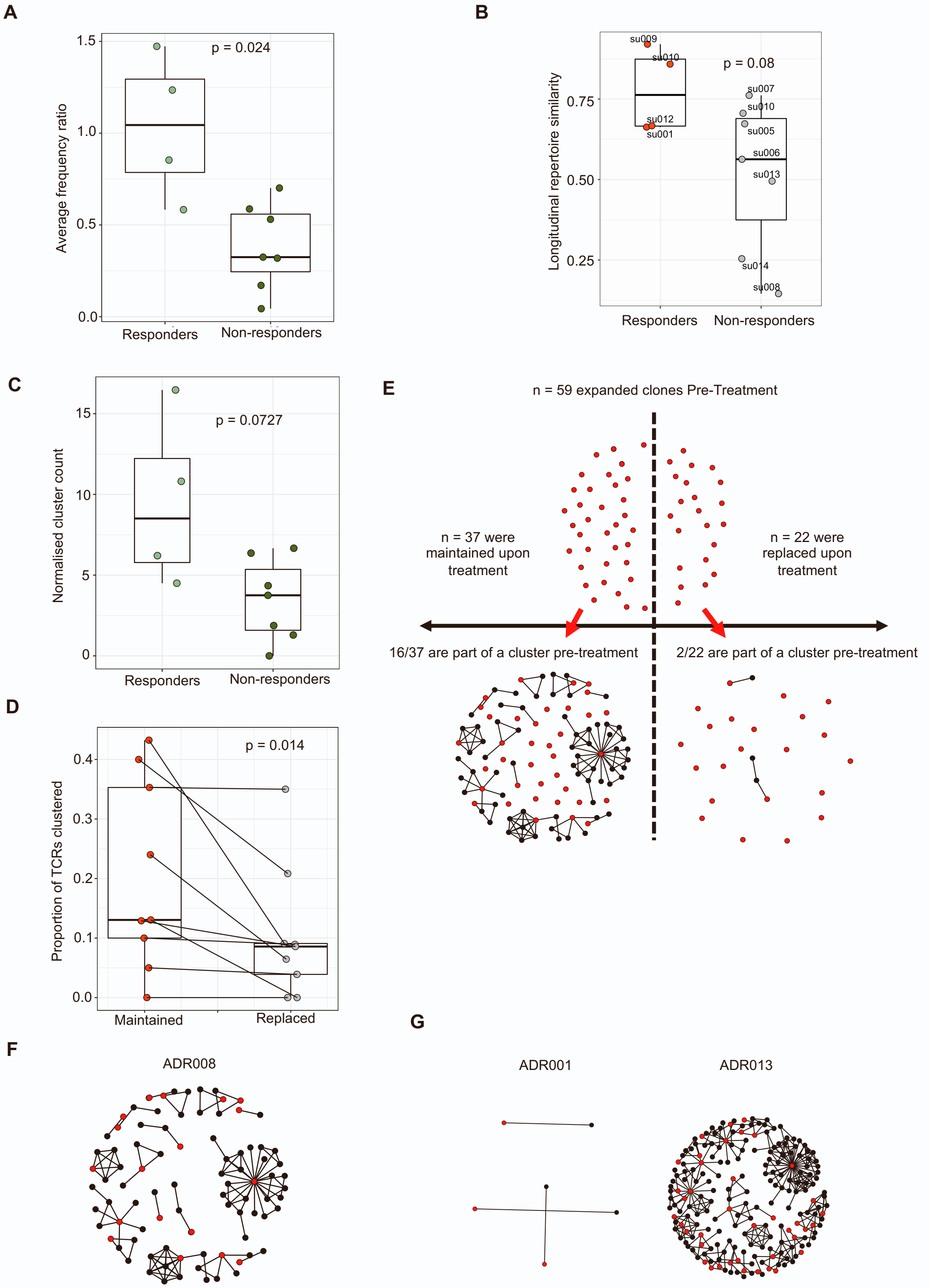

Figure S8. Additional expanded TCRs metrics, related to Figures 5 and 6

(A) The arithmetic mean of Pre/Post frequency ratios of clones expanded pre-treatment, per patient. Two-sided Mann–Whitney test *P* value shown. **(B)** Bulk TCR sequencing data from Yost et al. were retrieved from Adaptive Biotechnologies' ImmuneACCESS database (doi:10.21417/KY2019NM; https://clients.adaptivebiotech.com/pub/yost-2019-natmed). Intratumoural longitudinal similarity was measured with the cosine metric for 11 patients split between responders and non-responders as defined in Yost et al. Supplementary Table 1. One-sided Mann-Whitney P value is shown. **(C)** The post-treatment normalised number of clusters for the networks containing expanded sequences is shown. Two-sided Mann–Whitney test *P* value shown; n=11 patients. **(D)** The proportion of pre-treatment expanded TCRs that are part of a cluster as depicted in (C). TCRs were split between the ones that are also detected as expanded post-treatment and the ones that are not (respectively red circles and grey circles). Paired two-sided Mann–Whitney test *P* value shown. **(E)** Pre-treatment clustering around maintained and replaced expanded clones for ADR008. **(F)** Representative network diagrams of pre-treatment intratumoural CDR3 β-chain sequences for patient ADR008. The network shows sequences that are connected to at least one other TCR within the tumour. Clustering was performed around expanded intratumoral TCRs (red circles). **(G)** Representative network diagrams of post-treatment intratumoural CDR3 β-chain sequences for patient ADR001 (left) and for patient ADR013 (right). Clusters containing expanded sequences are shown.

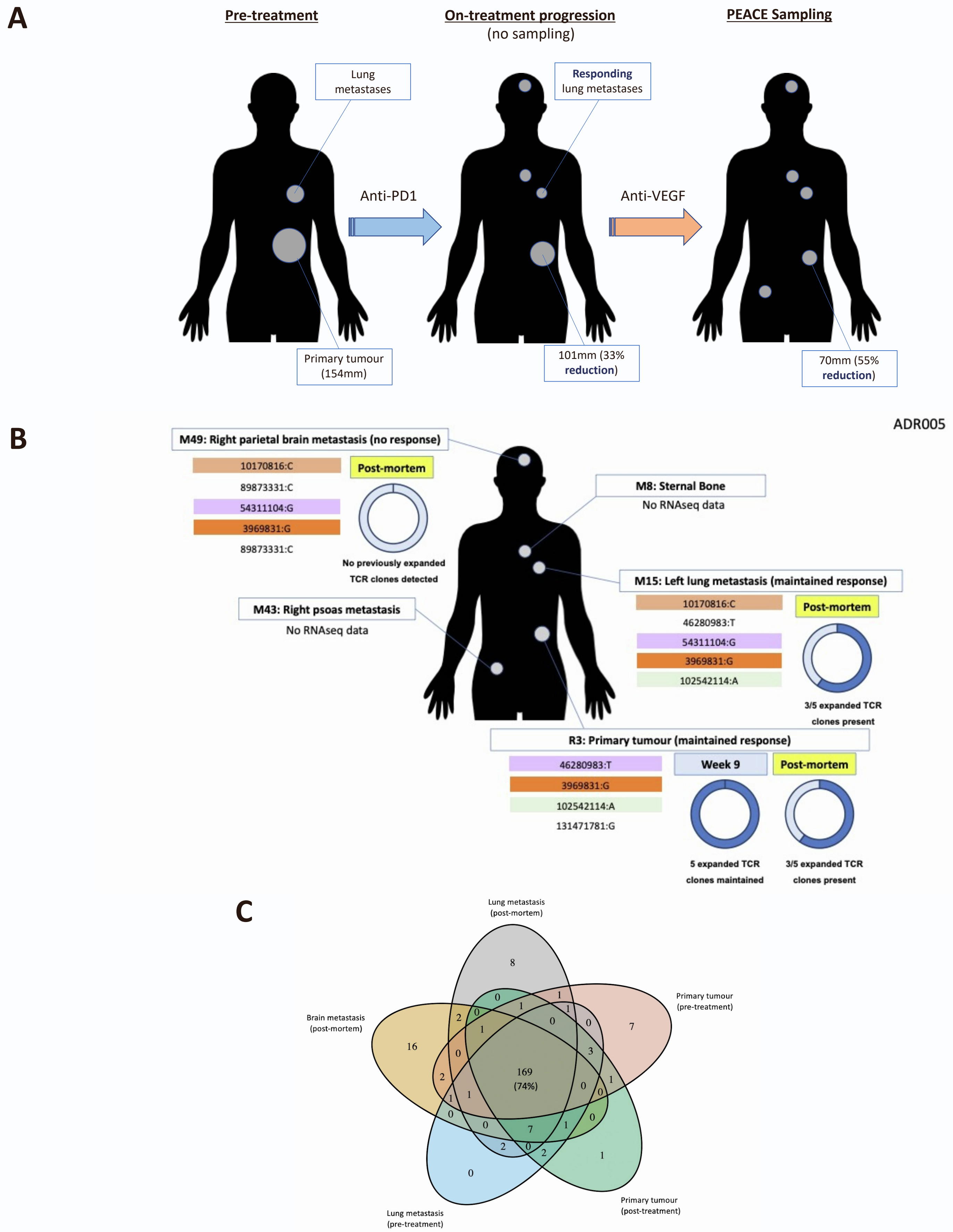

Figure S9. Schematic diagrams of ADR005 showing pre-/post-treatment, post-mortem sampling, and evolution of metastatic disease, related to Figure 5

(A) Clinical timeline for ADR005. **(B)** Post-mortem tumour samples are represented. The proportion of TCRs that were expanded both pre- and post-treatment during life (n=5) detected in each post-mortem sample, only samples where the detection rate is greater than 0 are displayed. 3/5 were detected in the lung metastatic and 1/5, 1/5, 2/5 and 3/5 were detected in region 1, region 2, region 3 and region 4 of the primary site, respectively. The median number of TCR sequences retrieved per post-mortem sample was 163 (range: 20-1340). Expressed mutations yielding predicted neoantigens are shown. Colour coding indicates sharing between sites. Non-coloured labels are private to the disease site. **(B)** Venn diagram showing number of mutations private and shared between primary tumour, lung (nivolumab-responsive) and brain (nivolumab-resistant) sites for ADR005. Biopsy context (pre/post-treatment or sampled post-mortem) are as labelled.

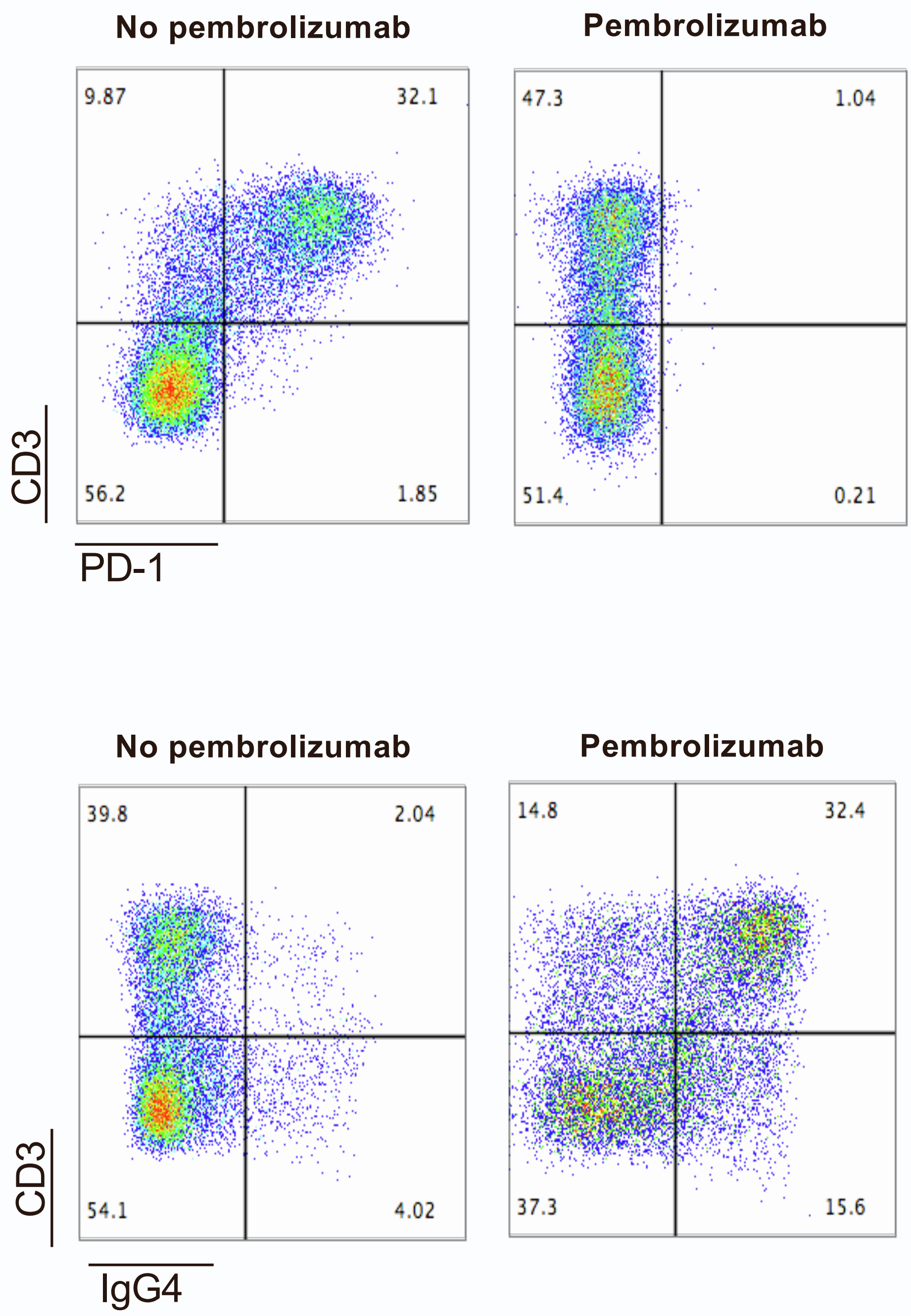

Figure S10. Competition assay with anti-PD1 antibody (pembrolizumab), related to Figure 6

In vitro assessment of activated PBMC demonstrates that PD-1 on T cells can be detected following pembrolizumab incubation using anti-human IgG4. **(A)** Incubation of activated PBMC with pembrolizumab blocks PD-1 flow cytometry staining (EH12.2 clone). **(B)** Pembrolizumab binding to PD-1 can be detected using an anti-IgG4 flow cytometry staining antibody. All dot plots are pre-gated on live single cells.

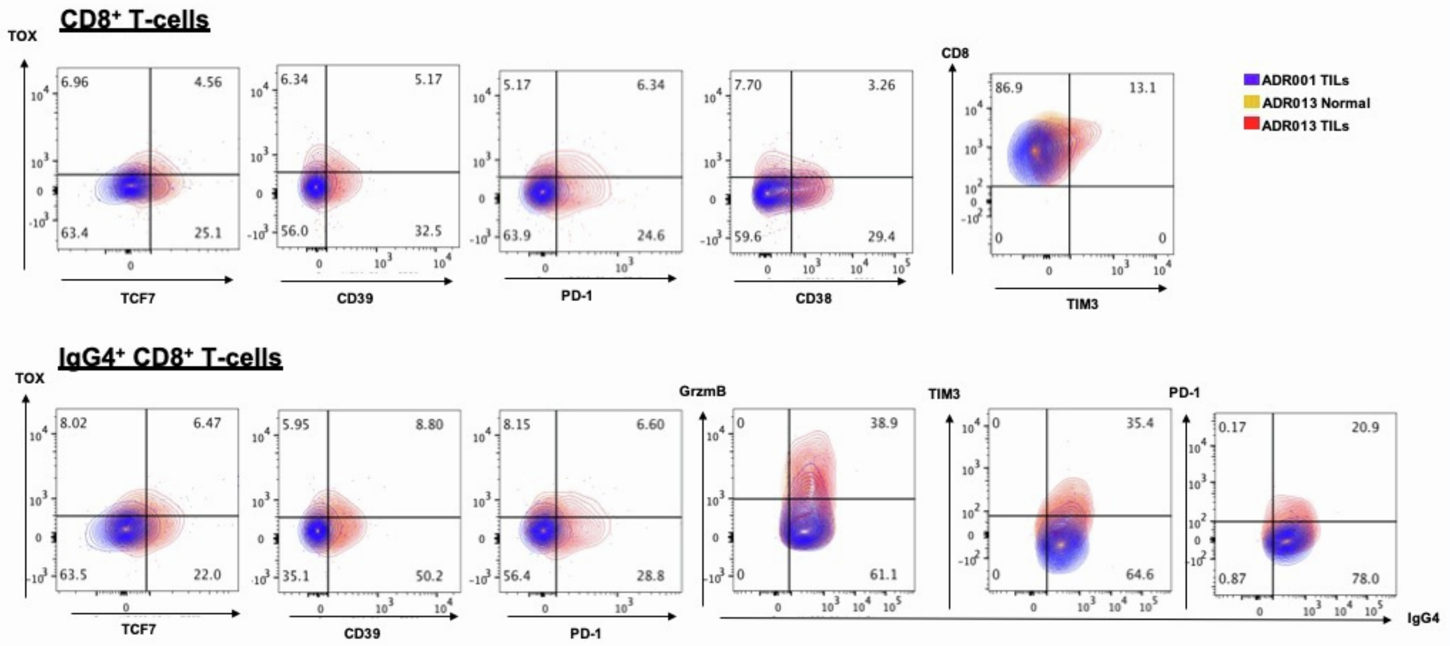

Figure S11. Flow cytometry-based analysis of ADR013 (responder) and ADR001 (non-responder) evaluating post-treatment total and nivolumab-bound CD8+ T-cells, related to Figure 6

FACS plots show the co-expression of markers on CD8+ and IgG4+CD8 cells in ADR001 TILs (tumour tissue), ADR013 TILs and ADR013 Normal (tumour-adjacent normal kidney tissue). *FACS - Fluorescence-activated cell sorting; TILs – tumour infiltrating lymphocytes*

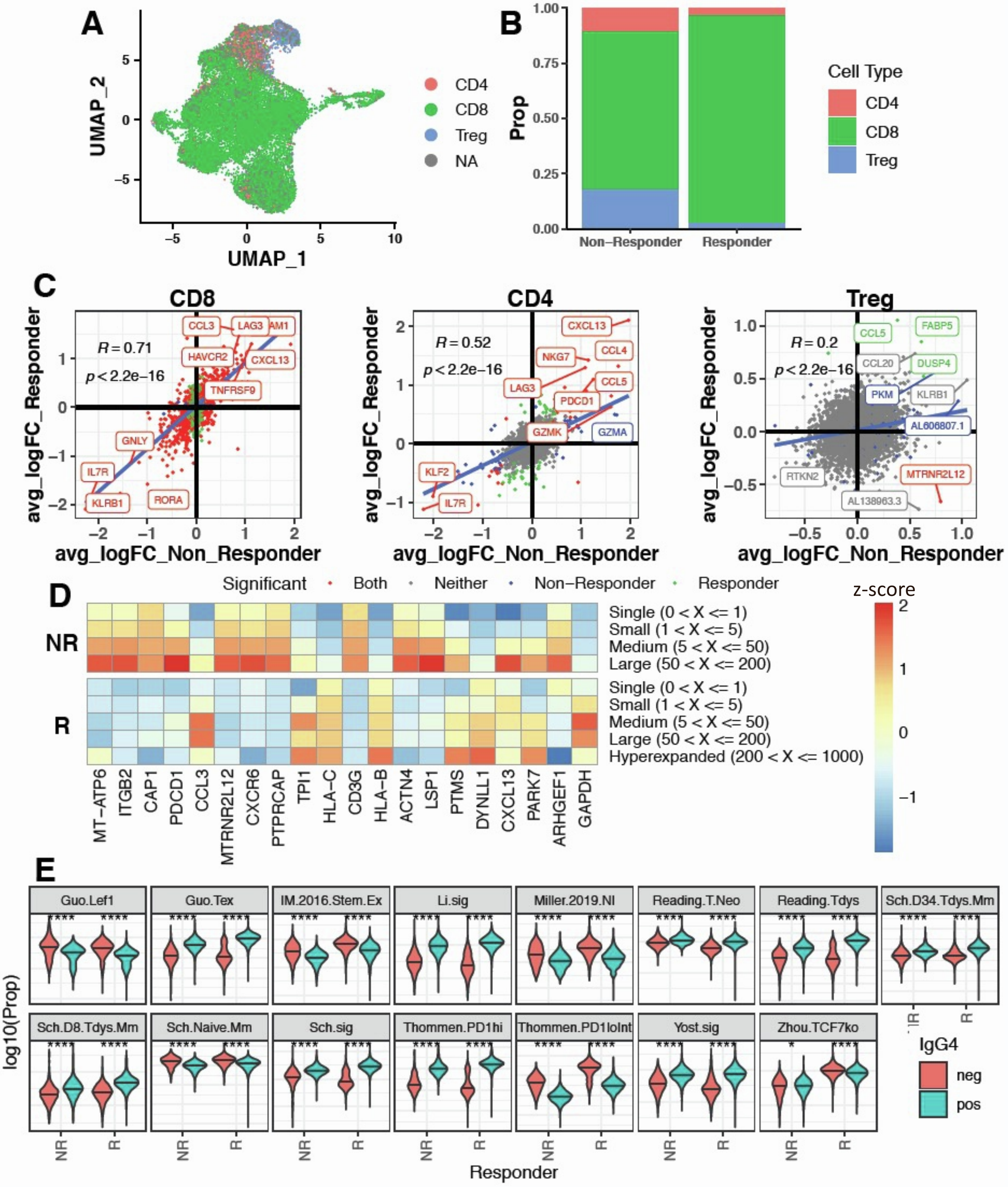

Figure S12. scRNA- and TCR-seq of ADR013 (responder) and ADR001 (non-responder), related to Figure 6
(A) UMAP of merged ADR001 (non-responder) and ADR013 (responder) scRNA data, coloured by cell type definition (CD8 = CD8+/CD4-/FOXP3-, CD4 effector = CD8-/CD4+/FOXP3-, Treg = CD8-/FOXP3+). **(B)** Proportions of each cell type recovered in each patient. **(C)** Differential gene expression analysis performed between IgG4^{+} and IgG4^{-} cells in each cell type for each patient, average logFC then plotted for responder vs non-responder. Regression line plotted using a linear model, colours indicate whether a logFC change was found significant in either or both patients. **(D)** Heatmaps showing top genes which positively correlated (Pearson's correlation) with TCR expansion in the non-responder (NR) patient. **(E)** Signature expression levels (calculated as the proportion of cell transcript mapping to genes in signature) by non-responder (NR) and responder (R) and IgG4 binding. Significance levels show the result of Wilcox test between IgG4 bound and unbound cells.

A **B**

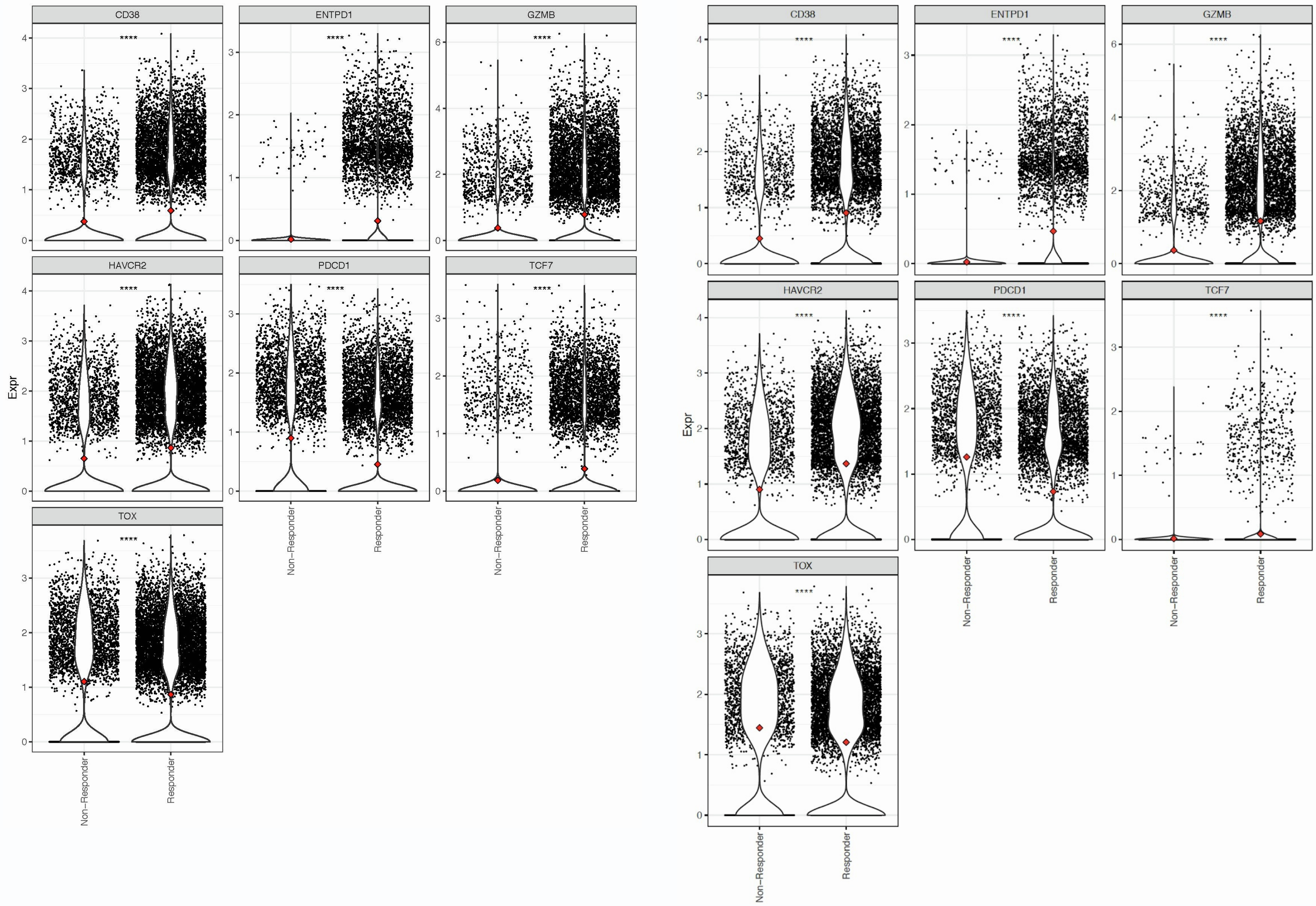

Figure S13. Single-cell gene expression analysis of CD8+ and IgG4+CD8+ T-cells, related to Figure 6
Single-cell RNAseq expression of Granzyme B, TCF7, TOX, HAVCR2 (TIM-3), CD38, ENTPD1(CD39) and PDCD1(PD-1) on **(A)** CD8+ and **(B)** IgG4+CD8+ T-cells in ADR013 (responder) and ADR001 (non-responder) are shown.

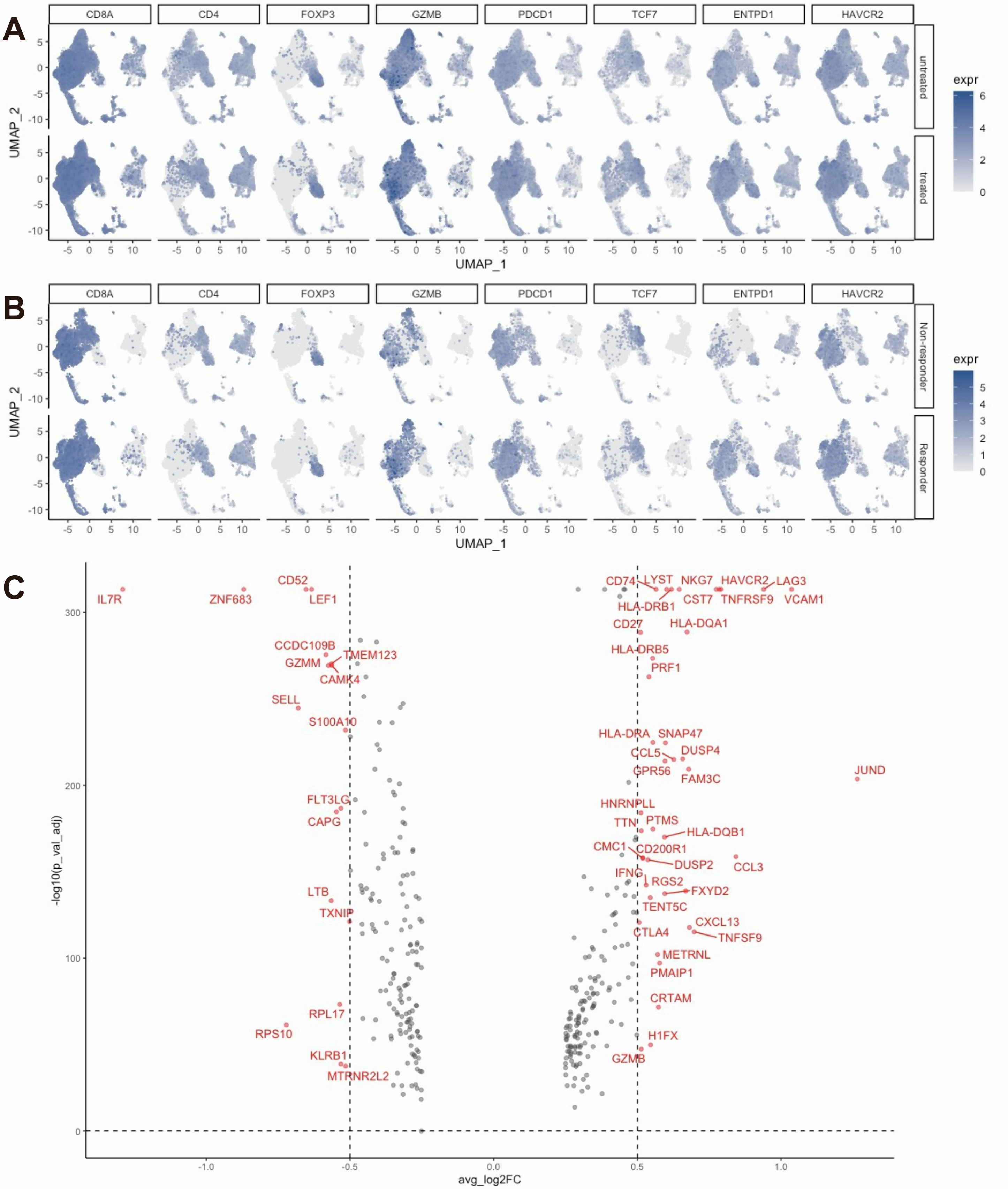

Figure S14. Single-cell RNAseq data across Braun *et al*., Krishna *et al*. Borcherding *et al.*, and ADAPTeR cohorts, related to Figure 7

(A) UMAP of single cell RNAseq data (scRNAseq) comparing expression of CD8, CD4, Tregs and Granzyme B, PDCD1, TCF7, ENTP1 and HAVCR2 on T-cell subsets in CPI treated and untreated samples across the Braun *et a*l., Krishna *et al*., Borcherding *et al.*, and ADAPTeR cohorts. (B) UMAP of scRNAseq data comparing expression of CD8, CD4, Tregs and Granzyme B, PDCD1, TCF7, ENTP1 and HAVCR2 on T-cell subsets in the responders and non-responder following CPI treatment using the Krishna *et al*. and ADAPTeR cohorts. Cell numbers were normalised between responders and non-responders. (C) Volcano plot shows the differential gene expression in singleton (left) and expanded (right) TCR clones in CPI treated and untreated samples (negative binomial Wald test, Benjamini-Hochberg corrected P values). Transcripts that were differentially regulated (FDR<0.05) and are labelled. *UMAP - Uniform Manifold Approximation and Projection; CPI – checkpoint inhibitor therapy.*
